# Genome-Wide Transcriptomic Analysis Identifies Pathways Regulated by Sterculic Acid in Retinal Pigmented Epithelium Cells

**DOI:** 10.3390/cells9051187

**Published:** 2020-05-11

**Authors:** Ana Pariente, Álvaro Pérez-Sala, Rodrigo Ochoa, Rafael Peláez, Ignacio M. Larráyoz

**Affiliations:** Center for Biomedical Research of La Rioja (CIBIR), Neurodegeneration Area, Biomarkers and Molecular Signaling Group, Piqueras 98, 26006 Logroño, Spain; apariente@riojasalud.es (A.P.); aperez@riojasalud.es (Á.P.-S.); rochoaf@riojasalud.es (R.O.); rpelaez@riojasalud.es (R.P.)

**Keywords:** sterculic acid, cell death, macular degeneration, genetic response

## Abstract

In addition to its predominant role in lipid metabolism and body weight control, *SCD1* has emerged recently as a potential new target for the treatment of various diseases. Sterculic acid (SA) is a cyclopropene fatty acid with numerous biological activities, generally attributed to its Stearoyl-CoA desaturase (SCD) inhibitory properties. Additional effects exerted by SA, independently of SCD inhibition, may be mediating anti-inflammatory and protective roles in retinal diseases such as age-related macular degeneration (AMD), but the mechanisms involved are poorly understood. In order to provide insights into those mechanisms, genome-wide transcriptomic analyses were carried out in mRPE cells exposed to SA for 24 h. Integrative functional enrichment analysis of genome-wide expression data provided biological insight about the protective mechanisms induced by SA. On the one hand, pivotal genes related to fatty acid biosynthesis, steroid biosynthesis, cell death, actin-cytoskeleton reorganization and extracellular matrix-receptor interaction were significantly downregulated by exposition to SA. On the other hand, genes related to fatty acid degradation and beta-oxidation were significantly upregulated. In conclusion, SA administration to RPE cells regulates crucial pathways related to cell proliferation, inflammation and cell death that may be of interest for the treatment of ocular diseases.

## 1. Introduction

The hormesis theory proposes that in early stages of damage, or after low intensity injuries, cells activate stress response mechanisms to promote cellular adaptation, repair, survival or even proliferation [1]. Nevertheless, high intensity injuries usually promote cell death through regulated or unregulated mechanisms such as apoptosis, necrosis, ferroptosis, pyroptosis, necroptosis, parthanatos, entosis or autopahagy [2,3]. The retina, like other tissues, must respond to environmental stimuli such as toxins, oxidative stress, inflammation, high metabolic activity or light-phototoxicity, which can induce deleterious effects over these Retinal Pigment Epithelium (RPE) cells [4,5]. Survival or death pathways activate depending on the stimuli, its intensity or the induced signaling.

The retina is a very complex tissue that transforms the visual information and transfers it to visual regions of the brain [6]. It is composed by more than 50 cell types organized in different structures. RPE, a polarized epithelial cell-layer located beneath the neuroretina, constitutes the outer blood-retinal barrier and provides nutrients to the photoreceptors [7,8]. RPE cell degeneration, or its anomalies, are found in multiple ocular abnormalities, including several macular dystrophies and age-related macular degeneration (AMD) [9], which is associated with formation of insoluble extracellular aggregates called drusen [4].

These deposits are composed of proteins, mineral and lipids [10], one which, 7-ketocholesterol (7KCh), is a central component associated with AMD pathology [11]. 7KCh is an oxysterol that has been related to several degenerative diseases, including AMD [12,13,14], atherosclerosis [15,16,17,18], Parkinson’s [19,20] and Alzheimer’s disease [21,22,23]. It has been demonstrated that 7KCh promotes an inflammatory and a cytotoxic response [14,24,25], but it also promotes cell arrest and cell death by different molecular mechanisms still not completely decoded [25]. This cholesterol-derived molecule increases the transcription and release of pro-inflammatory cytokine and growth factors such as IL-1β, IL-6, IL-8 and vascular endothelial growth factor (VEGF) [24,26], in NFκB, TLR4 and protein kinase-dependent manner [13,14,24].

Sterculic Acid (SA) is a cyclopropenoid fatty acid mainly obtained from the seeds of *Sterculia foetida* that have been demonstrated to counteract the inflammatory and cytotoxic responses caused by 7-Ketocholesterol (7KCh) in in vivo and in vitro models of choroidal neovascularization (CVN) [12]. It has been widely reported that SA is an in vivo and in vitro Stearoyl-CoA desaturase (SCD) inhibitor [27,28] through irreversible binding to the enzyme [29] or the transformation of SA into sterculoyl-Co [30]. SCD1 modulates the membrane-lipid composition as a result of its enzymatic activity [31] and modulates lipid metabolism and body weight control [32]. SCD1 has been demonstrated to have positive effects over palmitate-induced cytotoxicity in osteoclast and mesenchymal stromal cells [33]. SA treatments have also been demonstrated to reduce monounsaturated fatty acids (MUFAs) in bovine adipocytes cells while the total content in fatty acids has remained stable [34]. It has been also demonstrated that SA treatments, administrated as sterculic oil (SO), have many positive effects over different pathologies like glucose tolerance, blood pressure, body mass among others [27,35,36,37]. Inhibition of SCD1 by SA as a treatment for obesity has been also studied in Otsuka Long-Evans Tokushima Fatty rats [37].

Interestingly, lipid-reduction strategies are, nowadays, an alternative approach to prevent a variety of ocular diseases [38,39,40]. SA administration has been demonstrated to modify levels of lipogenic genes such as ACC, FAS, SREBP1a/c [34,41]. SA protective activity mechanisms against cell injuries are not completely understood. It was demonstrated that SA reduces the expression of some ER stress markers, such as C/EBP homologous protein (CHOP) and glucose-regulated protein, 78KDa (GRP78) [12] involving TLR4 receptor and interacts with many intracellular kinases in human RPE cells [13]. On the other hand, SCD1 inhibition could be an alternative pathway to mediate SA protective effects. SCD over-expression has been detected in many cancer types and it is related to increased cell proliferation, reduction of relapse-free survival and poor prognosis in patients [42] while its chemical inhibition, or gene silencing, reduces tumoral resistance and activates cell death pathways in many cancer cell lines [42]. Recently, it has been described that SCD1 inhibition promotes remyelination because, in macrophages, SCD1 controls cholesterol efflux transporters (ABCA1), increasing lipid accumulation and promoting a proinflammatory phenotype [43].

The molecular mechanism underlying the SA positive effects are still unknown. This study uses a genome-wide transcriptomic analysis to unveil the molecular pathways induced by SA treatments that protect RPE cells from cellular injuries. Our results demonstrated that the anti-inflammatory and protective effects of SA in RPE cells are not the result of SCD inhibition because SCD1 inhibition by CAY10566 does not protect RPE cells from the cytotoxic effects of 7KCh. Furthermore, transcriptomic signature differs completely from SA to CAY10566 indicating that most of the effects observed with SA treatment are independent of SCD inhibition.

## 2. Material and Methods

### 2.1. Cell Lines and Culture

ARPE19 and RF/6A cells were obtained from the ATCC (Manassas, VA 20108, USA). Monkey retinal pigment epithelium mRPE cells were a kind gift from Dr SP Becerra (National Eye Institute, NIH, Bethesda, MD). ARPE19 is a retinal pigment epithelia cell line derived from the normal eyes of a 19-year-old male. ARPE-19 has structural and functional properties characteristic of RPE cells in vivo and presents RPE-specific markers like CRALBP and RPE-65 [44]. mRPE cells were derived from Rhesus monkey eyes generated as described in [45]. These cells have been suggested to display a response to oxysterol challenge more physiologically relevant than what can be obtained using the ARPE-19 cell line [46]. RF/6A are chorioretinal endothelial cells widely used to model angiogenesis, differentiation and response drugs or environmental treatments in the choroid and retina [47]. The mRPE cell line was grown in DMEM/F12 1:1 medium (Hyclone-Thermo Scientific, Waltham, MA, USA) supplemented with 5% fetal bovine serum (Invitrogen, Alcobendas, Madrid, Spain) and supplemented with 1.5% of pyruvate, 1% of non-essential amino-acids and finally 1% penicillin/streptomycin (Hyclone-Thermo Scientific, Waltham, MA, USA). ARPE19 cell line was cultured in the same DMEM/F12 medium but supplemented with 10% fetal bovine serum. RF/6A cell line was grown in DMEM medium (ATCC, Manassas, VA 20108, USA) supplemented with 10% fetal bovine serum and 1% penicillin/streptomycin. Cultured cells were maintained in a 37 °C atmosphere containing 5% CO_2_ and 85% humidity.

### 2.2. Cell Treatments

Cells were seeded at a density of 100,000 cells/well in 12-well plates for MTT assays, or 1.25 × 10^6^ cells in P100 plates for RNA purification. Cells were allowed to attach for 24 h and cultured until 100% confluency. Then, serum-containing media was removed and changed to serum free medium for 24 h and more. After that, cells were treated for 24 h with different concentrations of 7KCh (Sigma-Aldrich, Steinheim, Germany) solved in β-Cyclodextrin (Sigma-Aldrich, Madrid, Spain) from 0 to 20 µM, Staurosporin from 0 to 1.5 µM (Abcam, Cambridge, UK), H_2_O_2_ from 0 to 2.75 mM (Sigma-Aldrich, Steinheim, Germany) for MTT assays. To study sterculic acid effects, cells were treated with sterculic acid dissolved in DMSO from 0 to 20 µM (PPQF, University of Alcalá, Spain) or with the SCD1 inhibitor CAY10556 (Abcam, Cambridge, UK) from 0 to 100 nM for 24 h.

### 2.3. Cell Viability Assays

Treatment toxicity or protective effect were analyzed after 24 h using the 3-(4,5-dimethylthiazol-2-yl)-5-(3-carboxymethoxyphenyl)-2-(4-sulfophenyl)-2H-tetrazolium bromide (MTS) assay (Promega, Madison, WI, USA). Cells were washed two times after 1:10 MTs-medium mixture adding, and data were measured after 4 h of incubation at 37 °C. Results are presented as percentage of viability over control vehicle treated cells.

### 2.4. RNA Purification

Total RNA was isolated from cell cultures using TRIzol (Invitrogen, Madrid, Spain), purified using the RNeasy mini-kit (Qiagen, Valencia, CA, USA), and treated with DNase I (Qiagen, Valencia, CA, USA) following the manufacturer’s instructions. 

### 2.5. Quantitative Real-Time PCR

One µg of total RNA was reverse-transcribed into first-strand cDNA using random primers and the SuperScript III kit (Invitrogen, Madrid, Spain) in a total volume of 20 μL according to the manufacturer’s instructions. The resulting cDNA was mixed with SYBR Green PCR Master Mix (Applied Biosystems, Carlsbad, CA) for quantitative real time polymerase chain reaction (qRT-PCR) using 0.3 µM forward and reverse oligonucleotide primers (Table 1). Quantitative measures were performed using a 7300 Real Time PCR System (Applied Biosystems, Madrid, Spain). Cycling conditions were an initial denaturation at 95 °C for 10 min, followed by 40 cycles of 95 °C for 15 s and 60 °C for 1 min. At the end, a dissociation curve was implemented from 60 to 95 °C to validate amplicon specificity. Gene expression was calculated using absolute quantification by interpolation into a standard curve. All values were divided by the expression of the house keeping gene 18. 

### 2.6. Next Generation Sequencing

Ultrasequencing were performed according to the manufacturer’s protocols and using their reagents (Illumina, San Diego, CA, USA) as described previously [48]. Briefly, the integrity and quality of total RNA were assessed with an automated electrophoresis system (Experion; Bio-Rad, Hercules, CA, USA). Then, mRNA was isolated from 1 μg of total RNA using poly-T oligonucleotide-attached magnetic beads. This mRNA was fragmented into approximately 200 base pair (bp) pieces by using divalent cations under elevated temperature. Cleaved RNA fragments were reverse transcribed into first strand cDNA using reverse transcriptase and random primers. Next, the second strand was synthesized using DNA polymerase I and RNase H. These double-stranded cDNA fragments were end-repaired by T4 DNA polymerase and Klenow DNA polymerase, phosphorylated by T4 polynucleotide kinase, and ligated to Illumina indexing adapters. These adapter-tagged libraries were amplified by using 15 cycles of PCR with DNA polymerase (Phusion; Finnzymes Reagents, Vantaa, Finland) and validated and quantified by electrophoresis and quantitative PCR (qPCR). Pools of six indexed libraries were mixed (multiplexed) at equimolar ratios to yield a total oligonucleotide mixture concentration of 10 nM. Finally, the resulting libraries were sequenced with the Genome Analyzer IIx platform (Illumina, Madrid, Spain) to generate 150-bp single reads. Six pooled indexed libraries were sequenced in each flow cell lane. Pathway alterations promoted by SA or CAY10556 treatment were studied using significant up and down regulated genes from high throughput sequencing. Cellular pathways differentially expressed were analyzed with specialized software (www.genemania.org; www.reactome.org) and shown with Kyoto Encyclopedia of Genes and Genomes (KEGG) [49,50]. In order to define which pathways were significantly modified by SA treatment a functional enrichment analysis was performed using a Pearson chi square test.

### 2.7. Inmunofluorescence and Confocal Microscopy

Cells were seeded onto 8-well NuncTM Lab-TekTM (ThermoFisher Scientific, Madrid, Spain) and grown and treated as previously described and cells were fixed with 4% PFA for 10 min. Afterwards, cells were permeabilized with 0.5% Triton ×100 in PBS for 5 min. Non-specific binding was blocked by incubation with 3% BSA (Sigma-Aldrich, Madrid, Spain) for 30 min at room temperature. Incubation with a specific primary antibody was carried out overnight with ZO-1 rabbit polyclonal antibody 1/500 (Thermo Fisher Scientific, Madrid, Spain). Samples were rinsed with PBS and subsequently incubated for 1 h at room temperature with Alexa-488 donkey anti-rabbit secondary antibody (LifeTechnologies, Oregon, USA) at a dilution of 1/800 and Hoechst (Sigma-Aldrich, Madrid, Spain) at 1/2000 *v/v*. Images were acquired in a confocal microscope (TCS SP5, Leica, Wetzlar, Germany).

### 2.8. ELISA

Levels of secreted VEGF, IL-6 in conditioned medium of mRPE cell cultures were measured 24 h after treatments with 15 μM 7KCh, SA 10 μM and combined treatments, using the Rhesus Macaque IL6 ELISA Kit (Raybiotech Life, Peachtree Corners, GA, USA) and the Monkey VEGFA ELISA Kit (MyBiosource, San Diego, CA, USA). ELISA results were quantified using the Biotek Synergy H4 multi-mode plate reader (BioTek Instruments, Covina, CA, USA).

### 2.9. Statistical Analysis

All data were analyzed with GraphPad Prism 6 software and were considered statistically significant when *p* < 0.05. Values are expressed as means ± SEM. Normally distributed data were evaluated by ANOVA followed by the Dunnet’s post-hoc test while data not following a normal distribution were analyzed with the Kruskal–Wallis test followed by the Mann–Whitney U test. Two-way ANOVA was used for multiple comparisons of independent means with SidaK modification.

## 3. Results

### 3.1. Sterculic Acid Does Not Present Cell Toxicity to Retinal Cell In Vitro

**Figure 1:**
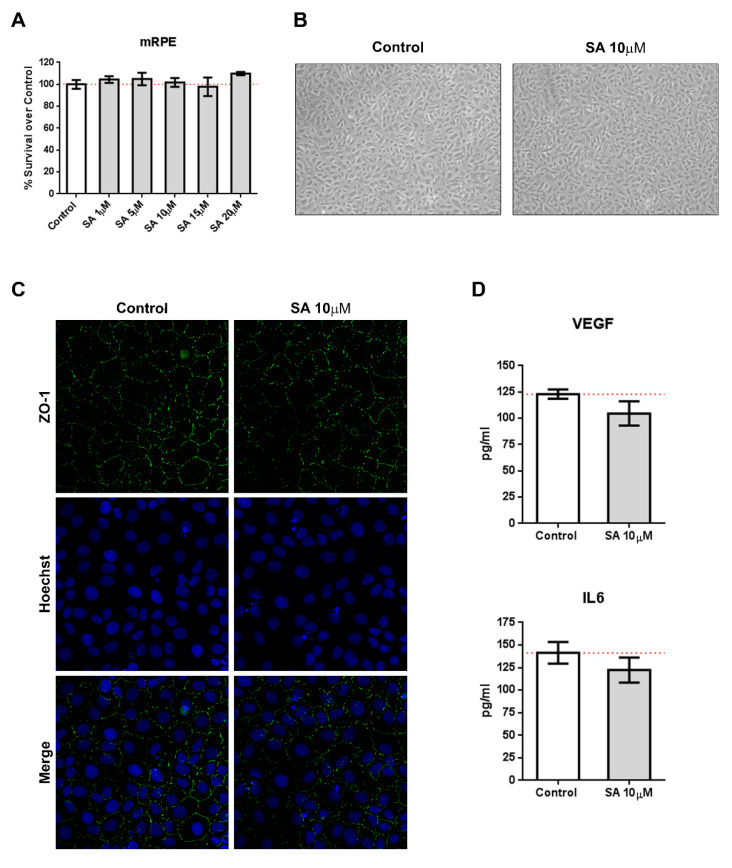
Sterculic acid (SA) does not show toxic effects in retinal cells. (**A**) Sterculic acid cytotoxicity (1–20 μM) in mRPE cells was measured by the MTS method. Data are presented as mean ± SEM of 12-well plates of four different experiments. The red dashed line is a guidance mark of 100% of viability in control cells. (**B**) Representative brightfield images of cell morphology in the control and 10 μM SA-treated mRPE cells after 24 h. (**C**) ZO-1 (green) distribution in cell-cell contacts of control and 10 μM SA-treated mRPE cells for 24 h. Cell nucleus (blue) was stained with the Hoechst DNA-marker. (**D**) VEFG an IL6 release was measured by ELISA in the conditioned media of the mRPE cells 24 h after treatment with or without 10 μM SA. The figures show mean ± SEM of at least three different experiments.

We incubated mRPE cells with increasing concentration of SA (1–20 μM) and performed MTT assays to test for toxicity. As it can be seen in Figure 1A, SA administration was not toxic for mRPE cells up to 20 μM. Identical experiments performed in ARPE-19 and RF/6A cells resulted in similar results (Figure S1). Likewise, SA administration did not induce inflammatory cytokines, such as VEGF or IL6, as assessed by ELISA (Figure 1D). Furthermore, brightfield images did not show any alterations in morphology (Figure 1B) after SA incubation and immunofluorescence for ZO-1 remained unchanged (Figure 1C).

### 3.2. Sterculic Acid Administration is Protective Against 7KCh-Induced Cell Death In Vitro

**Figure 2:**
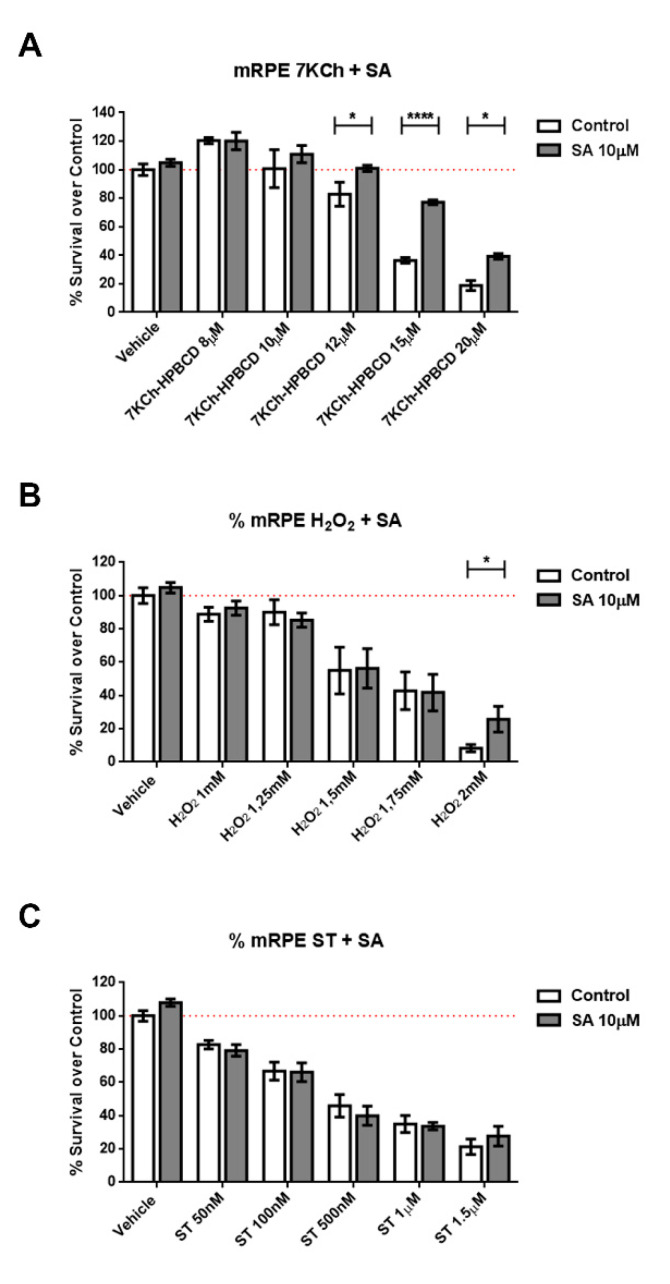
Protective effect of SA in different cell death models in mRPE retina cells. Cell survival in mRPE cell cultures was measured by the MTS method. (**A**) Protective effect over dose-dependent 7KCh-HPBCD cell death. (**B**) Protective effect over dose-dependent H_2_O_2_ oxidative stress injury. (**C**) Protective effect over dose-dependent caspase-3 dependent staurosporine cell death. Data are presented as mean ± SEM of 12-well plates of at least four different experiments. * *p* < 0.05, **** *p* < 0.0001. Red dashed line is a guidance mark of 100% of viability in control cells.

We subjected cells of retinal origin (mRPE, ARPE-19 and RF/6A cell lines) to three different cell death models: Staurosporine, (a well-known activator of caspase-3), hydrogen peroxide (oxidative stress) and 7KCh (apoptosis/necrosis mixed model). Co-incubation of cells with SA was able to reduce cell death induced by 7KCh (Figure 2 and Figure S2) in all cell lines. In the oxidative stress model (H_2_O_2_), protection by SA in mRPE cells was only observed at a concentration that killed 90% of cells, while in ARPE19 cells, SA was more effective. In addition, SA administration failed to protect cells from caspase3-mediated cell death (Figure 2 and Figure S2).

**Figure 3:**
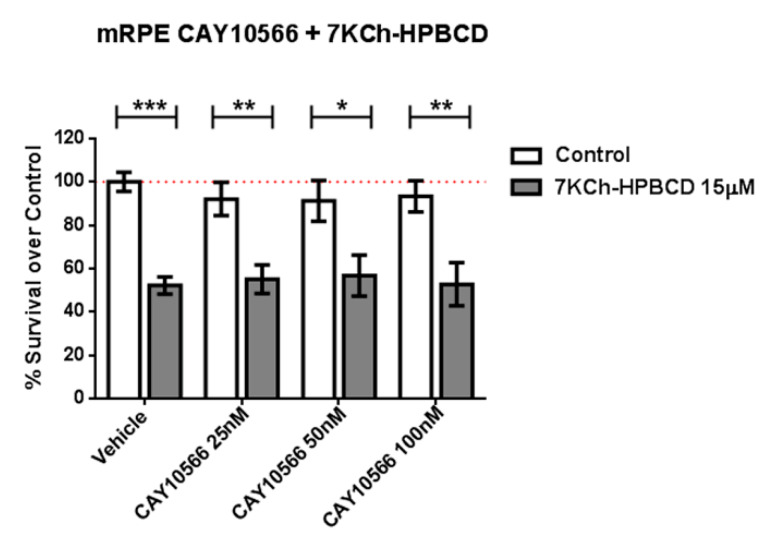
SCD1-inhibitor CAY10566 does not protect against the 7KCh-induced cell death in mRPE cells. Different CAY10566 doses were tested to restore cell viability against the 15 μM 7KCh-HPBCD cytotoxicity. Data represented mean ± SEM of 12-well plates of at least four different experiments. * *p* < 0.05, ** *p* < 0.01, *** *p* < 0.001. The red dashed line is a guidance mark of 100% of viability of control cells treated with vehicle only. White bars correspond to cells treated only with CAY10556 (0–100 nM), while grey bars correspond to cells treated with 7KCh-HPBCD 15 µM and CAY10556 (0–100 nM).

Because SA has been described as an SCD inhibitor (IC50 = 1 μM) [25,51] we tested whether the selective and potent SCD inhibitor CAY10566 (IC50 = 4–26 nM) was able to replicate the protective effects. We pre-treated mRPE cells with 25–100 nM CAY10566 for 2 h and performed MTT assays after administration of 15 μM 7KCh, which causes around 50% cell death in mRPE cells. As shown in Figure 3, 25–100 nM CAY10566 administration was not toxic for mRPE cells but it failed to protect against 7KCh-induced cell death.

**Figure 4:**
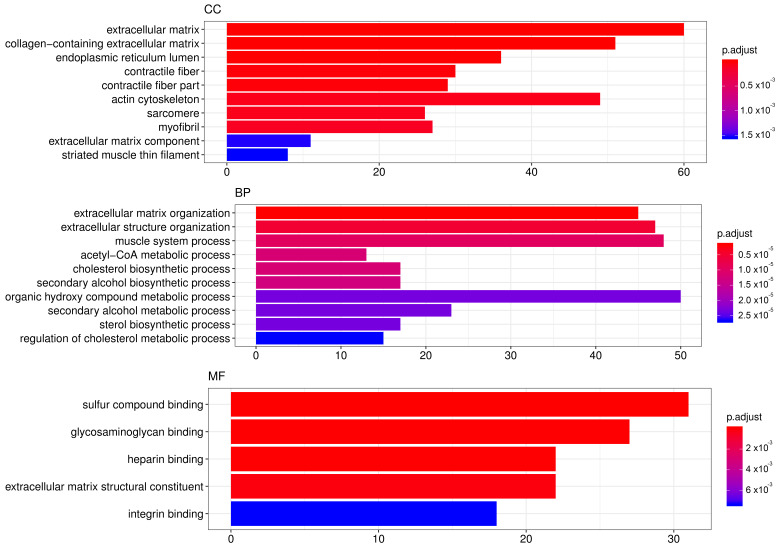
TOP GO categories for biological process, cellular component and molecular function in gene expression modified by SA. Gene ontology (GO) enrichment analysis was performed with DEGs induced by SA administration for 24 h to mRPE cells. The annotated DEGs were classified into the cellular component, molecular function and biological process categories by EnrichGO according to the GO terms. The y-axis shows the functional groups while the x-axis shows the number of genes in each category.

In order to elucidate which pathways were elicited by SA and may have been mediating its effects, we performed genome-wide transcriptome analysis in mRPE cells treated with 10 μM SA for 24 h. After applying FDR methods (Table S1), we obtained 922 differentially expressed genes (DEGs) by SA administration associated with a variety of biological processes and molecular functions (Figure 4, Table S1). TOP GO categories included extracellular matrix-related, actin-cytoskeleton-related and metabolic processes (Figure 4 and Table S2). 

Relevant genes associated with these pathways are shown in Table 2, Figures S3 and S4. On the one hand, pivotal genes related to fatty acid biosynthesis (*SREBF1*, *AAC1*, *FASN*, *SCD* and *CREB3L1*) as well as steroid biosynthesis (*HMGCS*, *HMGCR*, *MVD*, *FDFT1*, *SQLE*, *LSS*, *CYP51A1*, *MSMO1*, *HSD17B7*, *NSDHL*, *SC5D*, *EBP*) were significantly downregulated by exposition to SA. On the other hand, genes mediating fatty acid degradation and beta-oxidation were significantly upregulated (*CPT1A*, *SLC25A20*, *ACADS* and *ACADVL*). However, the highest number of DEGs were related to extracellular matrix (ECM)-Receptor interaction, cell adhesion and cell junction-related genes. SA markedly decrease the expression of collagens (*COL1A1*, *COL1A2*, *COL3A1*, *COL5A2*, *COL7A1*, *COL8A1*, *COL9A1*, *COl11A1*, *COL16A1*, *COL17A1*), laminins (*LAMA3*, *LAMC1*), trombospodins (*THBS3*), fibronectin, integrins (*ITGA5* and *ITGB2*), claudins (*CLDN16*), cadherins (*CDH1*, *CDH3*, *CDH10*, *CDH15*), *IGFBPs* (*IGFBP3*, *IGFBP4*, *IGFBP5*, *IGFBP8*, *IGFBP9*), syndecans (*SDC2*), *CD274*, versican and *NCAM1*, among others (Table 2 and Table S1). SA also decreased the expression of genes related to Actin-cytoskeleton reorganization (Filamin, Parvin, *MLC*, *MLCP* and *RHOJ*). Interestingly, cell death related genes (*CASP1*, *CASP8*, *GSDMD* and *TNFRSF10A*) were also downregulated by SA (Table 2 and Table S1).

**Figure 5:**
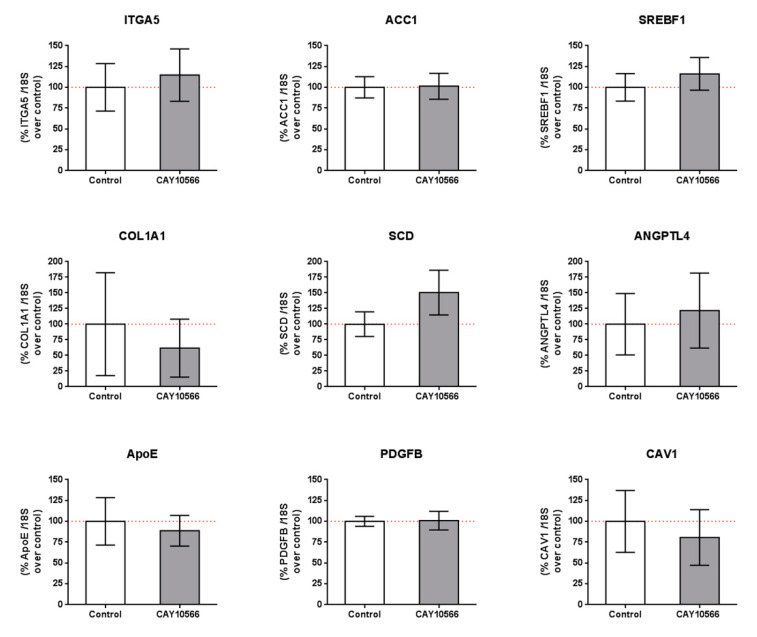
Gene expression in mRPE cells after 100 nM CAY10566 treatment for 24 h. A broad panel of genes related to cell adhesion, extracellular matrix, lipid synthesis and metabolism and cell signaling was checked. Data represent mean ± SEM gene expression with respect to the 18S-housekeeping gene of three different experiments. The Wilcoxon non-parametric test was used to evaluate related samples. The red dashed line is a guidance mark for 100% expression in control cells.

We also tested whether the observed changes in gene expression by SA treatment were due to SCD inhibition and/or other mechanisms. We incubated mRPE cells with 100 nM CAY10566 for 24 h and performed qPCR assays to measure gene expression of members of the pathways modified by SA (Table 2 and Table S1). mRPE cells incubated with 100 nM CAY10566 for 24 h did not show any change in the expression of those genes (Figure 5). RNASeq analyses of CAY10556-treated mRPE cells showed that SCD inhibition only modified the expression of 30 genes, of which only 10 were shared with SA treatment, thus confirming these results (Table S3).

Another pathway(s) induced by SA was related to *PPAR* signaling. We obtained a list of published, validated and computationally predicted, *PPAR* target genes (ppargene.org) and compared it to the list of DEGs induced by SA. From the 270 *PPAR* validated genes, 37 were modified by SA treatment (*p* < 0.0001). Likewise, from the 448 *PPAR* high confidence predicted genes, 58 were modified by SA treatment (*p* < 0.0001) including the *top 4* genes predicted with a confidence score of 1 (*PLIN2*, *SLC25A20*, *ANGPTL4*, *PDK4*).

## 4. Discussion

Sterculic acid (SA) is a cyclopropene fatty acid originally found in the seeds of the plant *Sterculia foetida* with numerous biological activities. SA is well-known because of its inhibitory effect on the activity of SCD, also known as ∆9-desaturase, both in vivo and in vitro [27,28,34,35,36,37,52]. Several authors have pointed out that it may be used as a coadjuvant in several pathologies such as nonalcoholic steatohepatitis, Alzheimer’s disease, cancer, and retinal disorders, where this enzyme has been associated [25,32,53,54,55,56]. Additionally, reports in the literature suggest additional effects independently of its SCD inhibitory properties with potential roles in inflammation, proliferation and cell death [12,13,25].

We decided to study the effects and mechanisms of action of SA in cells of retinal origin because of the potential therapeutic role of SA in ocular diseases [12,13,24]. We used a primary immortalized RPE cell line that recapitulates well RPE function and physiology [57,58], as well as ARPE19 and RF6/A cell lines to show lack of toxicity of SA up to 20 μM, which is a higher dose than the one used in vitro and in vivo to show effects [11,12,13,15,16,24,42]. These data were in agreement with reports showing the lack of toxicity in vivo, in hamsters or rats fed with an SO-enriched diet [27,41] or in vitro in differentiated adipocytes [34].

Once we determined the lack of toxicity of SA in culture, we tested the protective effects of SA incubation in three different models of cell death in vitro. We used hydrogen peroxide to mimic oxidative stress conditions, staurosporine, a caspase3-mediated apoptosis inducer compound and 7-ketocholesterol (7KCh), a toxic oxysterol that has been associated with AMD formation and development. SA did not protect against staurosporine-induced cell death in any cell line, indicating that SA administration cannot inhibit caspase3-mediated mode of cell death (Figure 2C, Figure S2E,F). However, SA was able to reduce H_2_O_2_-mediated cell death in mRPE, ARPE19 and RF6/A cell lines (Figure 2B, Figure S2C,D) and markedly reduce 7KCh-mediated cell death in all cell lines, especially those of pigment epithelium origin (Figure 2A, Figure S2A,B).

Given the SCD-inhibiting properties of SA, we then tested whether the protective effect was mediated by SCD inhibition. To our surprise, the potent, specific SCD inhibitor CAY10566 was not able to protect against 7KCh-induced cell death in mRPE cells (Figure 3) at concentrations well above its IC50, indicating that the protective effects observed with SA were not mediated by SCD inhibition but by, at least mostly, other mechanisms.

In order to elucidate the mechanisms exerted by SA in mRPE, independently of SCD inhibition, we performed genome-wide transcriptome analysis in mRPE cells treated with 10 μM SA for 24 h. We obtained 922 differentially expressed genes (DEGs) associated with a variety of biological processes and molecular functions after SA treatment. The most relevant pathways were related to ECM molecule secretion, cell adhesion, metabolism and cell death (Table 2). Interestingly, many of the changes induced by SA administration were independent of SCD inhibition (Figure 5) because CAY10566 failed to reproduce those changes in mRPE cells. These changes include genes related to ECM-receptor interaction (*ITGA5*, *COL1A1* and *CAV1*), fatty acid and cholesterol metabolism (*ACC1*, *SREBF1*, *APOE*) as well as angiogenic genes (*ANGPTL4* and *PDGFB*) and even SCD. CAY10566 has been shown to inhibit the activity of SCD without altering its expression up to 48 h supporting our findings that SA effect on SCD expression is independent of its SCD inhibitory effect, at least in the short term [51].

There are several reports in the literature describing the role of increased SCD1 activity in different diseases [27,36,52,59], especially in those associated with lipid metabolism and metabolic syndrome [27,36,41]. Beneficial effects include the improvement of serum levels of triglycerides and adiponectin and glucose tolerance and the reduction of weight and blood pressure [27,35,37,60]. However, side effects have been also reported, including hypercholesterolemia, problems in reproduction or carcinogenesis due to SCD activity inhibition [41,61,62,63]. 

One of the main alterations in mRPE cells after SA treatment is in gene expression related to fatty acid and cholesterol metabolism (Figure 4, Table 2 and Table S1). We report a generalized decrease in genes regulating crucial steps in fatty acid synthesis (*ACC1*, *SREBF1*, *FASN*, *SCD* and *CRE3L1*) and cholesterol metabolism (*HMGCS*, *HMGCR*, *MVD*, *FDFT1*, *SQLE*, *LSS*, *CYP51A1*, *MSMO1*, *HSD17B7*, *NSDHL*, *SC5D*, *EBP*) which has been attributed before to SA treatment. Thus, in bovine adipocytes, treatment with SA decreased the expression of ACC1 [34]. In hamster liver, ACC, FAS and SREBF1 hepatic mRNA levels were decreased after sterculic oil supplementation [41]. Likewise, in Otsuka Long–Evans Tokushima Fatty rats, FAS and SREBF1 mRNA levels were also reduced when SA was administered [27]. The role of CAY10566 in the regulation of genes related to fatty acid and cholesterol metabolism is controversial. For instance, in pig embryos CAY10566 administration resulted in a decrease in SREBF1, ARF1, PLD1 and ERK2 [58] while in MAC-T cells CAY10566 reduced SCD, SREBF1, ACSS2 and LPIN1 mRNA levels [57]. In our experiments, with mRPE cells, changes in the expression of genes regulating fatty acid and cholesterol seem to be independent of SCD inhibition because CAY10566 failed to change the expression of genes such as *ACC1*, *SCD*, *SREBF1* (Figure 5), *HMGCR* (data not shown) or *ACC2* (data not shown).

In addition to a decrease in fatty acid biosynthesis-related genes, changes induced by SA showed an increase in genes regulating beta-oxidation. SA induced the expression of *CPT1A*, *SLC25A20*, *ACADS* and *ACADVL* as it occurs in bovine adipocytes treated with SA [34] as a consequence of a reduction in the levels of ACC that would provoke the increase of the mitochondrial fatty acid oxidation. Additionally, in agreement with our results, other genes related to cholesterol metabolism such as *LDLR*, *PPARs* and *SREBP2* remained unchanged [41]. 

Other genes regulated by SA, particularly relevant for the treatment of retinal disorders, are those related to angiogenesis. Thus, SA decreased the expression of *PDGFB* and *ANGPTL4.* In addition, it reduced the expression of genes related to endothelial proliferation including *FGF7*, *FGF10*, *PDGFB* and others, which may be of relevance to decreasing the angiogenesis observed in some ocular disorders. In this sense, 7KCh is an oxysterol that has been related to several degenerative diseases, including AMD formation and development. Interestingly, SA was able to reduce many genes involved in sterol biosynthesis, cell death (*CASP1*, *CASP8*, *GSDMD*), PPAR and TLR signaling pathways, which may explain why it is particularly effective against 7KCh-induced inflammation and cell death both in vivo and in vitro [12,13,14]. The reduction in the sterol biosynthesis pathways may regulate the intracellular metabolism of 7KC, while the regulation of the TLR pathways may decrease the transport of the oxysterol inside the cell. Additionally, the reduction in cell death mediators and the regulation of PPAR-related members may be a general effect decreasing inflammation and toxicity.

In conclusion, SA administration to RPE cells regulates crucial pathways for retinal cell proliferation, survival, inflammation and cell death that may be of interest in use as a coadjuvant of several pathologies. In addition to SCD inhibition, it seems to exert most of its effects independently of its SCD inhibitory properties, which is particularly interesting in the treatment of retinal diseases because side effects could be greatly reduced by performing topic administrations.

## Figures and Tables

**Table 1:** List of primers used in this study.

Gene Name	Oligonucleotide Sequence
SCD-F	5′-ATAAGTTGGAGACGACGCCC-3′
SCD-R	5′-GGCTCCCAAGTGTAGCAGAG-3′
SREBF1-F	5′-CGTTTCTTCGTGGATGGGGA-3′
SREBF1-R	5′-TTCAGTGCTCGCTCCAAGAG-3′
ITGA5-F	5′-TCTTGCTGGACTGTGGAGAG-3′
ITGA5-R	5′-AGGGCATTCTTGTCACCCAG-3′
APOE-F	5′-CTGCGTTGCTGGTCACATTC-3′
APOE-R	5′-CGCAGGTAATCCCAAAAGCG-3′
ANGPTL4-F	5′-CAAGGCTCAGAACAGCAGGA-3′
ANGPTL4-R	5′-CTCTTTCTTCGGGCAGGCTT-3′
CAV1-F	5′-GCAGAACCAGAAGGGACACA-3′
CAV1-R	5′-GATGCCAAAGAGGGCAGACA-3′
PDGFB-F	5′-CCACTCCATCCGCTCCTTC-3′
PDGFB-R	5′-CTCCTTCTTCCACGAGCCAG-3′
COL1A1-F	5′-GCCAAGACGAAGACATCCCA-3′
COL1A1-R	5′-GGCAGTTCTTGGTCTCGTCA-3′
ACC1-F	5′-ATTGCCTTCATGGGTCCTCC-3′
ACC1-R	5′-CTCCAGGGAAGAGTTGGGAT-3′
18S-F	5′-ATGCTCTTAGCTGAGTGTCCCG-3′
18S-R	5′-ATTCCTAGCTGCGGTATCCAGG-3′

**Table 2:** Relevant pathways and associated genes modified by 10 μM SA incubated for 24 h in mRPE cells.

Pathway	Gene	Log2 Fold Change	FDR	Gene Description
Fatty acid biosynthesis				
	*CREB3L1*	−0.68	1.29 × 10^−2^	cAMP responsive element binding protein 3 like 1
	*SREBF1*	−0.79	1.83 × 10^−2^	sterol regulatory element binding transcription factor 1
	*FASN*	−0.62	2.18 × 10^−2^	fatty acid synthase
	*ACC1*	−0.47	6.86 × 10^−6^	Acetyl-CoA Carboxylase Alpha
	*SCD*	−1.18	3.18 × 10^−5^	Stearoyl-CoA Desaturase
Steroid Biosynthesis				
	*HMGCS1*	−0.87	1.13 × 10^−2^	3-Hydroxy-3-Methylglutaryl-CoA Synthase
	*MVD*	−0.66	3.06 × 10^−2^	mevalonate diphosphate decarboxylase
	*FDFT1*	−0.31	1.14 × 10^−3^	Farnesyl-Diphosphate Farnesyltransferase 1
	*SQLE*	−0.50	1.41 × 10^−5^	squalene epoxidase
	*LSS*	−0.77	3.89 × 10^−6^	lanosterol synthase
	*CYP51A1*	−0.47	2.18 × 10^−2^	Cytochrome P450 Family 51 Subfamily A Member 1
	*MSMO1*	−0.61	1.35 × 10^−2^	Methylsterol Monooxygenase 1
	*HSD17B7*	−0.28	3.60 × 10^−2^	3-keto-steroid reductase
	*NSDHL*	−0.53	3.74 × 10^−3^	NAD(P) Dependent Steroid Dehydrogenase-Like
	*EBP*	−0.75	4.75 × 10^−7^	EBP Cholestenol Delta-Isomerase
Fatty acid degradation and beta-oxidation				
	*CPT1A*	0.97	1.07 × 10^−12^	Carnitine Palmitoyltransferase 1A
	*SLC25A20*	0.82	4.61 × 10^−6^	solute carrier family 25 member 20
	*ACADS*	0.50	5.76 × 10^−3^	acyl-CoA dehydrogenase short chain
	*ACADVL*	0.58	1.63 × 10^−5^	acyl-CoA dehydrogenase very long chain
Cell Death				
	*CASP8*	−0.36	3.20 × 10^−2^	caspase 8
	*CASP1*	−0.86	3.38 × 10^−2^	caspase-1
	*GSDMD*	−0.59	2.21 × 10^−3^	gasdermin D
	*TNFRSF10A*	−0.52	6.56 × 10^−4^	TNF receptor superfamily member 10a
ECM-Receptor interaction, cell adhesion, Cell junction				
	*COL1A1*	−1.37	9.76 × 10^−5^	collagen type I alpha 1 chain
	*COL1A2*	−0.96	2.61 × 10^−7^	collagen type I alpha 2 chain
	*COL3A1*	−1.66	2.74 × 10^−21^	collagen type III alpha 1 chain
	*COL5A2*	−0.73	3.23 × 10^−20^	collagen type V alpha 2 chain
	*COL7A1*	−1.04	2.25 × 10^−5^	Collagen Type VII Alpha 1 Chain
	*COL8A1*	−0.62	3.38 × 10^−5^	collagen type VIII alpha 1 chain
	*COL9A1*	−2.11	1.27 × 10^−4^	Collagen Type IX Alpha 1 Chain
	*COl11A1*	−0.63	1.45 × 10^−4^	collagen type XI alpha 1 chain
	*COL16A1*	−1.82	2.78 × 10^−10^	collagen type XVI alpha 1 chain
	*COL17A1*	−2.91	9.06 × 10^−4^	collagen type XVII alpha 1 chain
	*LAMA3*	−0.98	8.32 × 10^−7^	laminin subunit alpha 3
	*LAMC1*	−0.32	2.90 × 10^−2^	laminin subunit gamma 1
	*THBS3*	−0.50	1.73 × 10^−2^	thrombospondin 3
	*FN1*	−0.86	4.27 × 10^−4^	fibronectin 1
	*CHST2*	1.14	3.64 × 10^−5^	carbohydrate sulfotransferase 2
	*CHST12*	0.68	4.09 × 10^−2^	carbohydrate sulfotransferase 12
	*CHST15*	0.56	6.66 × 10^−4^	carbohydrate sulfotransferase 15
	*CD274*	−0.82	1.39 × 10^−2^	CD274 Molecule
	*ITGA5*	−0.64	1.29 × 10^−2^	integrin subunit alpha 5
	*ITGB2*	−0.36	1.41 × 10^−2^	integrin subunit beta 2
	*CLDN16*	−1.46	3.74 × 10^−2^	claudin 16
	*IGFBP3*	−0.93	2.69 × 10^−6^	insulin like growth factor binding protein 3
	*IGFBP4*	−0.59	1.50 × 10^−2^	insulin like growth factor binding protein 4
	*IGFBP5*	−2.31	1.62 × 10^−50^	insulin like growth factor binding protein 5
	*IGFBP8*	1.01	4.49 × 10^−5^	insulin like growth factor binding protein 8
	*IGFBP9*	−1.74	8.74 × 10^−08^	insulin like growth factor binding protein 9
	*SDC2*	−0.44	7.97 × 10^−3^	syndecan 2
	*CDH1*	−1.38	4.37 × 10^−4^	cadherin 1
	*CDH3*	−2.29	3.50 × 10^−15^	cadherin 3
	*CDH10*	−1.50	1.60 × 10^−2^	cadherin 10
	*CDH15*	−1.22	4.74 × 10^−2^	cadherin 15
	*VCAN*	−1.18	1.14 × 10^−2^	versican
	*NCAM1*	−0.27	3.20 × 10^−3^	neural cell adhesion molecule 1
Actin cytoskeleton reorganization				
	*FLNB*	−0.48	4.16 × 10^−2^	filamin B
	*PARVA*	−0.33	2.97 × 10^−4^	parvin alpha
	*MYLPF*	−1.31	1.05 × 10^−3^	myosin light chain, phosphorylatable, fast skeletal muscle
	*MLCP*	−0.59	2.39 × 10^−2^	Protein Phosphatase 1 Regulatory Subunit 12A
	*RHOJ*	−1.21	1.32 × 10^−2^	ras homolog family member J

## Supplementary Materials

The following are available online at https://www.mdpi.com/2073-4409/9/5/1187/s1, Figure S1: Sterculic acid does not show toxic effects in retinal cell lines, Figure S2: Protective effect of SA over cytotoxic agents on mRPE retinal cells, Figure S3: Steroid biosynthesis pathways is altered by 10 μM SA treatment in mRPE cells, Figure S4. Circular plot representing main pathways altered by 10 μM SA treatment in mRPE cells, Table S1: DEGs in mRPE cells after SA treatment, Table S2: GO categories of DEGs in mRPE cells after SA treatment, Table S3: DEGs in mRPE cells after CAY10556 treatment.

## Abbreviations

7KCh	7-ketocholesterol
AMD	Age-related macular degeneration
CVN	Choroidal neovascularization
DEG	Differentially expressed genes
DMEM	Dulbecco’s Modified Eagle medium
ECM	Extracellular matrix
ER	Endoplasmic reticulum
FDR	False discovery rate
GO	Gene ontology
HPBCD	Hydroxypropyl-beta-cyclodextrin
KEGG	Kyoto Encyclopedia of Genes and Genomes
mRPE	Monkey retinal pigment epithelium cells
MUFA	Monounsaturated fatty acid
RPE:	Retinal pigment epithelium
SA	Sterculic acid
SO	Sterculic oil

## References

[1] Mattson M.P. (2008). Hormesis defined. Ageing Res. Rev..

[2] Galluzzi L., Vitale I., Aaronson S.A., Abrams J.M., Adam D., Agostinis P., Alnemri E.S., Altucci L., Amelio I., Andrews D.W. (2018). Molecular mechanisms of cell death: Recommendations of the Nomenclature Committee on Cell Death 2018. Cell Death Differ..

[3] Ashkenazi A., Salvesen G. (2014). Regulated cell death: Signaling and mechanisms. Annu. Rev. Cell Dev. Biol..

[4] Bird A.C. (2010). Therapeutic targets in age-related macular disease. J. Clin. Investig..

[5] Chistyakov D.V., Baksheeva V.E., Tiulina V.V., Goriainov S.V., Azbukina N.V., Gancharova O.S., Arifulin E.A., Komarov S.V., Chistyakov V.V., Tikhomirova N.K. (2020). Mechanisms and Treatment of Light-Induced Retinal Degeneration-Associated Inflammation: Insights from Biochemical Profiling of the Aqueous Humor. Int. J. Mol. Sci..

[6] Grossniklaus H.E., Geisert E.E., Nickerson J.M. (2015). Introduction to the Retina. Prog. Mol. Biol. Transl. Sci..

[7] Bhutto I., Lutty G. (2012). Understanding age-related macular degeneration (AMD): Relationships between the photoreceptor/retinal pigment epithelium/Bruch’s membrane/choriocapillaris complex. Mol. Aspects Med..

[8] Strauss O. (2005). The retinal pigment epithelium in visual function. Physiol. Rev..

[9] Ambati J., Fowler B.J. (2012). Mechanisms of age-related macular degeneration. Neuron.

[10] Thompson R.B., Reffatto V., Bundy J.G., Kortvely E., Flinn J.M., Lanzirotti A., Jones E.A., McPhail D.S., Fearn S., Boldt K. (2015). Identification of hydroxyapatite spherules provides new insight into subretinal pigment epithelial deposit formation in the aging eye. Proc. Natl. Acad. Sci. USA.

[11] Moreira E.F., Larrayoz I.M., Lee J.W., Rodriguez I.R. (2009). 7-Ketocholesterol is present in lipid deposits in the primate retina: Potential implication in the induction of VEGF and CNV formation. Investig. Ophthalmol. Vis. Sci..

[12] Huang J.D., Amaral J., Lee J.W., Larrayoz I.M., Rodriguez I.R. (2012). Sterculic acid antagonizes 7-ketocholesterol-mediated inflammation and inhibits choroidal neovascularization. Biochim. Biophys. Acta.

[13] Huang J.D., Amaral J., Lee J.W., Rodriguez I.R. (2014). 7-Ketocholesterol-induced inflammation signals mostly through the TLR4 receptor both in vitro and in vivo. PLoS ONE.

[14] Rodriguez I.R., Larrayoz I.M. (2010). Cholesterol oxidation in the retina: Implications of 7KCh formation in chronic inflammation and age-related macular degeneration. J. Lipid Res..

[15] Buttari B., Segoni L., Profumo E., D’Arcangelo D., Rossi S., Facchiano F., Businaro R., Iuliano L., Rigano R. (2013). 7-Oxo-cholesterol potentiates pro-inflammatory signaling in human M1 and M2 macrophages. Biochem. Pharmacol..

[16] Hayden J.M., Brachova L., Higgins K., Obermiller L., Sevanian A., Khandrika S., Reaven P.D. (2002). Induction of monocyte differentiation and foam cell formation in vitro by 7-ketocholesterol. J. Lipid Res..

[17] Pedruzzi E., Guichard C., Ollivier V., Driss F., Fay M., Prunet C., Marie J.C., Pouzet C., Samadi M., Elbim C. (2004). NAD(P)H oxidase Nox-4 mediates 7-ketocholesterol-induced endoplasmic reticulum stress and apoptosis in human aortic smooth muscle cells. Mol. Cell Biol..

[18] Vejux A., Lizard G. (2009). Cytotoxic effects of oxysterols associated with human diseases: Induction of cell death (apoptosis and/or oncosis), oxidative and inflammatory activities, and phospholipidosis. Mol. Aspects Med..

[19] Cheng D., Jenner A.M., Shui G., Cheong W.F., Mitchell T.W., Nealon J.R., Kim W.S., McCann H., Wenk M.R., Halliday G.M. (2011). Lipid pathway alterations in Parkinson’s disease primary visual cortex. PLoS ONE.

[20] Nakaya K., Ayaori M., Uto-Kondo H., Sotherden G.M., Nishida T., Katamoto H., Miura Y., Takiguchi S., Yakushiji E., Iizuka M. (2013). Overexpression of stearoyl-coenzyme A desaturase 1 in macrophages promotes reverse cholesterol transport. Biochim. Biophys. Acta.

[21] Olivier E., Dutot M., Regazzetti A., Leguillier T., Dargere D., Auzeil N., Laprevote O., Rat P. (2016). P2X7-pannexin-1 and amyloid beta-induced oxysterol input in human retinal cell: Role in age-related macular degeneration?. Biochimie.

[22] Phan H.T., Hata T., Morita M., Yoda T., Hamada T., Vestergaard M.C., Takagi M. (2013). The effect of oxysterols on the interaction of Alzheimer’s amyloid beta with model membranes. Biochim. Biophys. Acta.

[23] Testa G., Staurenghi E., Zerbinati C., Gargiulo S., Iuliano L., Giaccone G., Fanto F., Poli G., Leonarduzzi G., Gamba P. (2016). Changes in brain oxysterols at different stages of Alzheimer’s disease: Their involvement in neuroinflammation. Redox Biol..

[24] Larrayoz I.M., Huang J.D., Lee J.W., Pascual I., Rodriguez I.R. (2010). 7-ketocholesterol-induced inflammation: Involvement of multiple kinase signaling pathways via NFkappaB but independently of reactive oxygen species formation. Investig. Ophthalmol Vis. Sci..

[25] Pariente A., Pelaez R., Perez-Sala A., Larrayoz I.M. (2019). Inflammatory and cell death mechanisms induced by 7-ketocholesterol in the retina. Implications for age-related macular degeneration. Exp. Eye Res..

[26] Dugas B., Charbonnier S., Baarine M., Ragot K., Delmas D., Menetrier F., Lherminier J., Malvitte L., Khalfaoui T., Bron A. (2010). Effects of oxysterols on cell viability, inflammatory cytokines, VEGF, and reactive oxygen species production on human retinal cells: Cytoprotective effects and prevention of VEGF secretion by resveratrol. Eur. J. Nutr..

[27] Ortinau L.C., Nickelson K.J., Stromsdorfer K.L., Naik C.Y., Pickering R.T., Haynes R.A., Fritsche K.L., Perfield J.W. (2013). Sterculic oil, a natural inhibitor of SCD1, improves the metabolic state of obese OLETF rats. Obesity (Silver Spring).

[28] Bichi E., Toral P.G., Hervas G., Frutos P., Gomez-Cortes P., Juarez M., de la Fuente M.A. (2012). Inhibition of 9-desaturase activity with sterculic acid: Effect on the endogenous synthesis of cis-9 18:1 and cis-9, trans-11 18:2 in dairy sheep. J. Dairy Sci..

[29] Raju P.K., Reiser R. (1967). Inhibition of fatty acyl desaturase by cyclopropene fatty acids. J. Biol. Chem..

[30] Jeffcoat R., Pollard M.R. (1977). Studies on the inhibition of the desaturases by cyclopropenoid fatty acids. Lipids.

[31] Galbraith L., Leung H.Y., Ahmad I. (2018). Lipid pathway deregulation in advanced prostate cancer. Pharmacol. Res..

[32] Uto Y. (2016). Recent progress in the discovery and development of stearoyl CoA desaturase inhibitors. Chem. Phys. Lipids.

[33] Dalla Valle A., Vertongen P., Spruyt D., Lechanteur J., Suain V., Gaspard N., Brion J.P., Gangji V., Rasschaert J. (2019). Induction of Stearoyl-CoA 9-Desaturase 1 Protects Human Mesenchymal Stromal Cells Against Palmitic Acid-Induced Lipotoxicity and Inflammation. Front. Endocrinol. (Lausanne).

[34] Kadegowda A.K., Burns T.A., Pratt S.L., Duckett S.K. (2013). Inhibition of stearoyl-CoA desaturase 1 reduces lipogenesis in primary bovine adipocytes. Lipids.

[35] Gomez F.E., Bauman D.E., Ntambi J.M., Fox B.G. (2003). Effects of sterculic acid on stearoyl-CoA desaturase in differentiating 3T3-L1 adipocytes. Biochem. Biophys. Res. Commun..

[36] Herrera-Meza M.S., Mendoza-Lopez M.R., Garcia-Barradas O., Sanchez-Otero M.G., Silva-Hernandez E.R., Angulo J.O., Oliart-Ros R.M. (2013). Dietary anhydrous milk fat naturally enriched with conjugated linoleic acid and vaccenic acid modify cardiovascular risk biomarkers in spontaneously hypertensive rats. Int. J. Food Sci. Nutr..

[37] Ortinau L.C., Pickering R.T., Nickelson K.J., Stromsdorfer K.L., Naik C.Y., Haynes R.A., Bauman D.E., Rector R.S., Fritsche K.L., Perfield J.W. (2012). Sterculic Oil, a Natural SCD1 Inhibitor, Improves Glucose Tolerance in Obese ob/ob Mice. ISRN Endocrinol..

[38] Pikuleva I.A., Curcio C.A. (2014). Cholesterol in the retina: The best is yet to come. Prog. Retin. Eye Res..

[39] Fliesler S.J., Ferrington D.A. (2019). EDITORIAL: Special issue on the role of lipid and protein oxidation in retinal degenerations. Exp. Eye Res..

[40] Fliesler S.J., Xu L. (2018). Oxysterols and Retinal Degeneration in a Rat Model of Smith-Lemli-Opitz Syndrome: Implications for an Improved Therapeutic Intervention. Molecules.

[41] Major C.A., Ryan K., Bennett A.J., Lock A.L., Bauman D.E., Salter A.M. (2008). Inhibition of stearoyl CoA desaturase activity induces hypercholesterolemia in the cholesterol-fed hamster. J. Lipid Res..

[42] Pelaez R., Pariente A., Perez-Sala A., Larrayoz I.M. (2020). Sterculic Acid: The Mechanisms of Action beyond Stearoyl-CoA Desaturase Inhibition and Therapeutic Opportunities in Human Diseases. Cells.

[43] Bogie J.F.J., Grajchen E., Wouters E., Corrales A.G., Dierckx T., Vanherle S., Mailleux J., Gervois P., Wolfs E., Dehairs J. (2020). Stearoyl-CoA desaturase-1 impairs the reparative properties of macrophages and microglia in the brain. J. Exp. Med..

[44] Dunn K.C., Aotaki-Keen A.E., Putkey F.R., Hjelmeland L.M. (1996). ARPE-19, a human retinal pigment epithelial cell line with differentiated properties. Exp. Eye Res..

[45] Becerra S.P., Fariss R.N., Wu Y.Q., Montuenga L.M., Wong P., Pfeffer B.A. (2004). Pigment epithelium-derived factor in the monkey retinal pigment epithelium and interphotoreceptor matrix: Apical secretion and distribution. Exp. Eye Res..

[46] Pfeffer B.A., Xu L., Porter N.A., Rao S.R., Fliesler S.J. (2016). Differential cytotoxic effects of 7-dehydrocholesterol-derived oxysterols on cultured retina-derived cells: Dependence on sterol structure, cell type, and density. Exp. Eye Res..

[47] Songstad A.E., Worthington K.S., Chirco K.R., Giacalone J.C., Whitmore S.S., Anfinson K.R., Ochoa D., Cranston C.M., Riker M.J., Neiman M. (2017). Connective Tissue Growth Factor Promotes Efficient Generation of Human Induced Pluripotent Stem Cell-Derived Choroidal Endothelium. Stem Cells Transl. Med..

[48] Larrayoz I.M., Rua O., Velilla S., Martinez A. (2014). Transcriptomic profiling explains racial disparities in pterygium patients treated with doxycycline. Investig. Ophthalmol. Vis. Sci..

[49] Kanehisa M., Goto S. (2000). KEGG: Kyoto encyclopedia of genes and genomes. Nucleic Acids Res..

[50] Kanehisa M., Goto S., Sato Y., Kawashima M., Furumichi M., Tanabe M. (2014). Data, information, knowledge and principle: Back to metabolism in KEGG. Nucleic Acids Res..

[51] Ralston J.C., Badoud F., Cattrysse B., McNicholas P.D., Mutch D.M. (2014). Inhibition of stearoyl-CoA desaturase-1 in differentiating 3T3-L1 preadipocytes upregulates elongase 6 and downregulates genes affecting triacylglycerol synthesis. Int. J. Obes. (Lond.).

[52] Hao P., Alaraj I.Q., Dulayymi J.R., Baird M.S., Liu J., Liu Q. (2016). Sterculic Acid and Its Analogues Are Potent Inhibitors of Toxoplasma gondii. Korean J. Parasitol..

[53] Astarita G., Jung K.M., Vasilevko V., Dipatrizio N.V., Martin S.K., Cribbs D.H., Head E., Cotman C.W., Piomelli D. (2011). Elevated stearoyl-CoA desaturase in brains of patients with Alzheimer’s disease. PLoS ONE.

[54] Fritz V., Benfodda Z., Rodier G., Henriquet C., Iborra F., Avances C., Allory Y., de la Taille A., Culine S., Blancou H. (2010). Abrogation of de novo lipogenesis by stearoyl-CoA desaturase 1 inhibition interferes with oncogenic signaling and blocks prostate cancer progression in mice. Mol. Cancer Ther..

[55] Hess D., Chisholm J.W., Igal R.A. (2010). Inhibition of stearoylCoA desaturase activity blocks cell cycle progression and induces programmed cell death in lung cancer cells. PLoS ONE.

[56] Scaglia N., Igal R.A. (2008). Inhibition of Stearoyl-CoA Desaturase 1 expression in human lung adenocarcinoma cells impairs tumorigenesis. Int. J. Oncol..

[57] Wang T., Lee H., Zhen Y. (2018). Responses of MAC-T Cells to Inhibited Stearoyl-CoA Desaturase 1 during cis-9, trans-11 Conjugated Linoleic Acid Synthesis. Lipids.

[58] Lee D.K., Choi K.H., Hwang J.Y., Oh J.N., Kim S.H., Lee C.K. (2019). Stearoyl-coenzyme A desaturase 1 is required for lipid droplet formation in pig embryo. Reproduction.

[59] Seibert J.T., Abuajamieh M., Sanz Fernandez M.V., Johnson J.S., Kvidera S.K., Horst E.A., Mayorga E.J., Lei S., Patience J.F., Ross J.W. (2018). Effects of heat stress and insulin sensitizers on pig adipose tissue. J. Anim. Sci..

[60] Herrera-Meza S., Rodriguez-Landa J.F., Martinez A.J., Herrera-Meza G., Fernandez-Demeneghi R., Reyes-Saldana K., Oliart-Ros R.M. (2017). Behavioral Effect of Sterculia apetala Seed Oil Consumption in Male Zucker Rats. J. Med. Food.

[61] Allen E., Johnson A.R., Fogerty A.C., Pearson J.A., Shenstone F.S. (1967). Inhibition by cyclopropene fatty acids of the desaturation of stearic acid in hen liver. Lipids.

[62] Lee D.J., Wales J.H., Sinnhuber R.O. (1971). Promotion of aflatoxin-induced hepatoma growth in trout by methyl malvalate and sterculate. Cancer Res..

[63] Lock A.L., Corl B.A., Barbano D.M., Bauman D.E., Ip C. (2004). The anticarcinogenic effect of trans-11 18:1 is dependent on its conversion to cis-9, trans-11 CLA by delta9-desaturase in rats. J. Nutr..

