# Supplementary material for: Genome-Wide Transcriptomic Analysis Identifies Pathways Regulated by Sterculic Acid in Retinal Pigmented Epithelium Cells
Source: Cells. 2020 May 11;9(5):1187. doi: 10.3390/cells9051187 (PMC7290791; doi:10.3390/cells9051187)
Supplement: Supplementary file 1 [file cells-09-01187-s001.pdf]

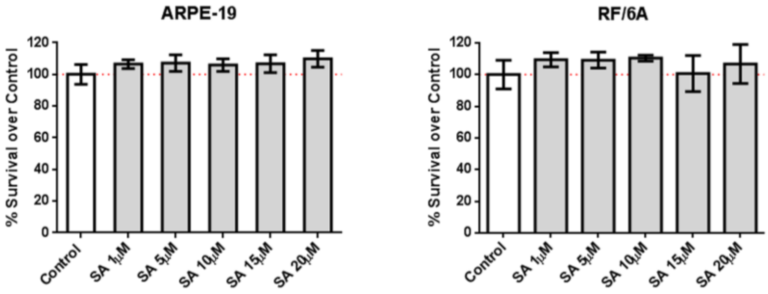

Supplementary Figure 1. Sterculic acid does not show toxic effects in retinal cell lines**.** Sterculic Acid cytotoxicity (1μM–20μM) in the ARPE19 and RF/6A cells measured by the MTS method. Data are presented as mean±SEM of 12−well plates and 4 different experiments. Red dashed line is a guidance mark of 100% of viability over control.

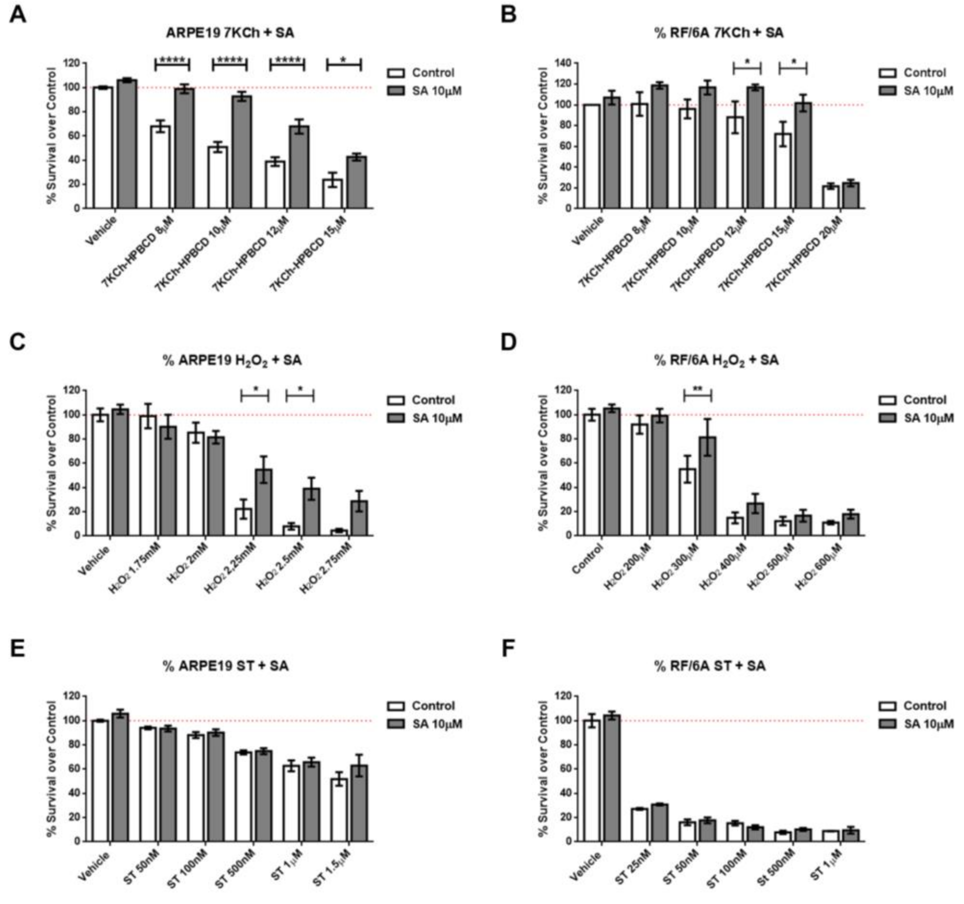

Supplementary Figure 2. Protective effect of SA over cytotoxicitic agents on mRPE retinal cells. Cell survival result of 10 μM SA treatment in mRPE cells was measured by the MTS method. Protective effect over dose−dependent pro−inflamatory 7KCh cell death in ARPE19 (**A**) and RF/6A cells (**B**). Protective effect over dose−dependent H_2O_2 oxidative stress injury in ARPE19 (**C**) and RF/6A cells (**D**). Protective effect over dose−dependent caspase−3 dependent staurosporine cell death in ARPE19 (**E**) and RF/6A cells (**F**). Data are presented as mean ± SEM of 12−well plates of 4 different experiments. *$P < 0.05$, **$P < 0.01$, ****$P < 0.0001$. Red dashed line is a guidance mark of 100 % of viability of control cells.

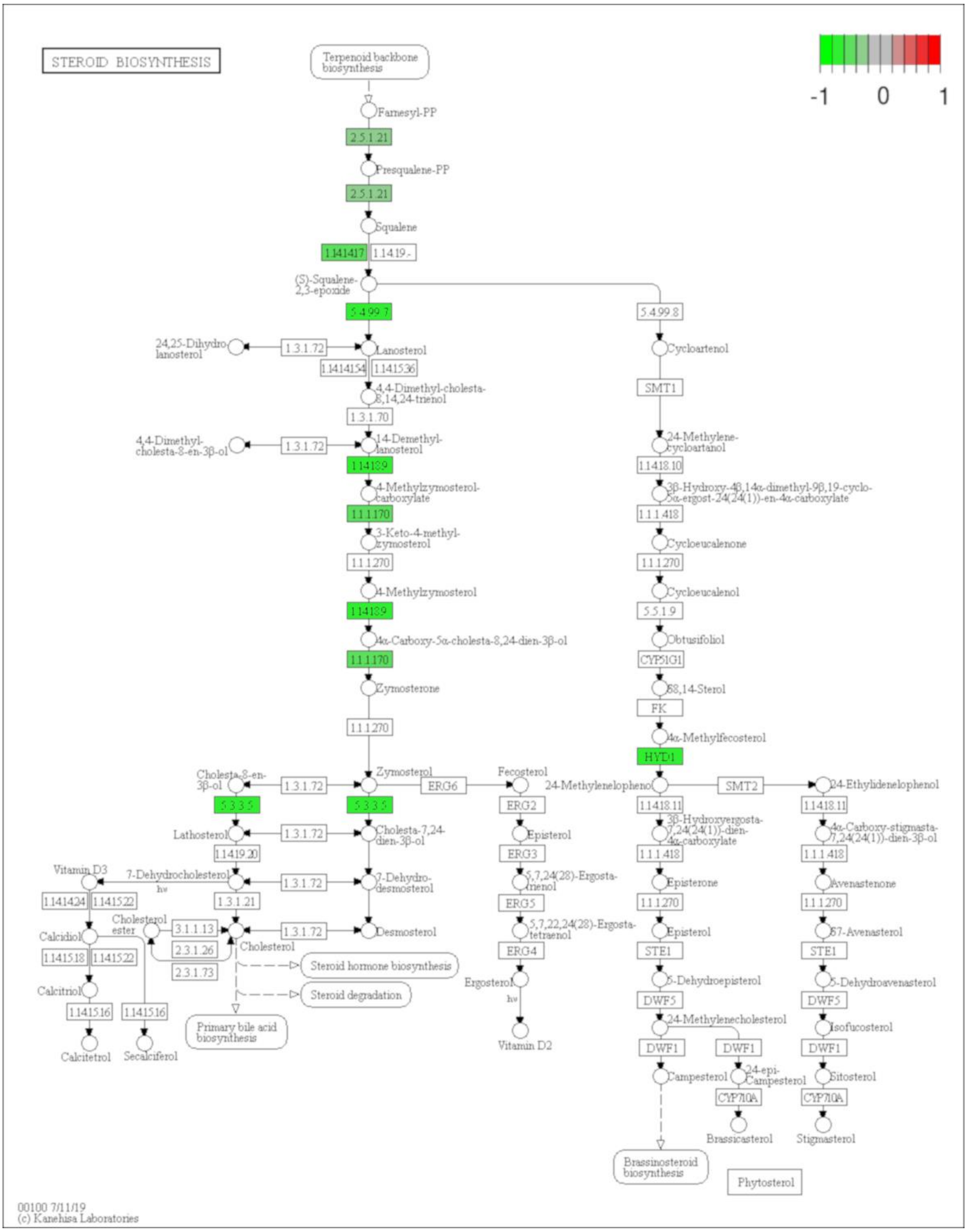

Supplementary Figure 3. Steroid biosynthesis pathways is altered by 10 μM SA treatment in mRPE cells. Proteins in red are expressed at higher levels while proteins in green are expressed at lower levels than controls. Reprinted with permission from Kyoto Encyclopedia of Genes and Genomes (KEGG). Copyright 2013, KEGG, Kyoto, Japan.

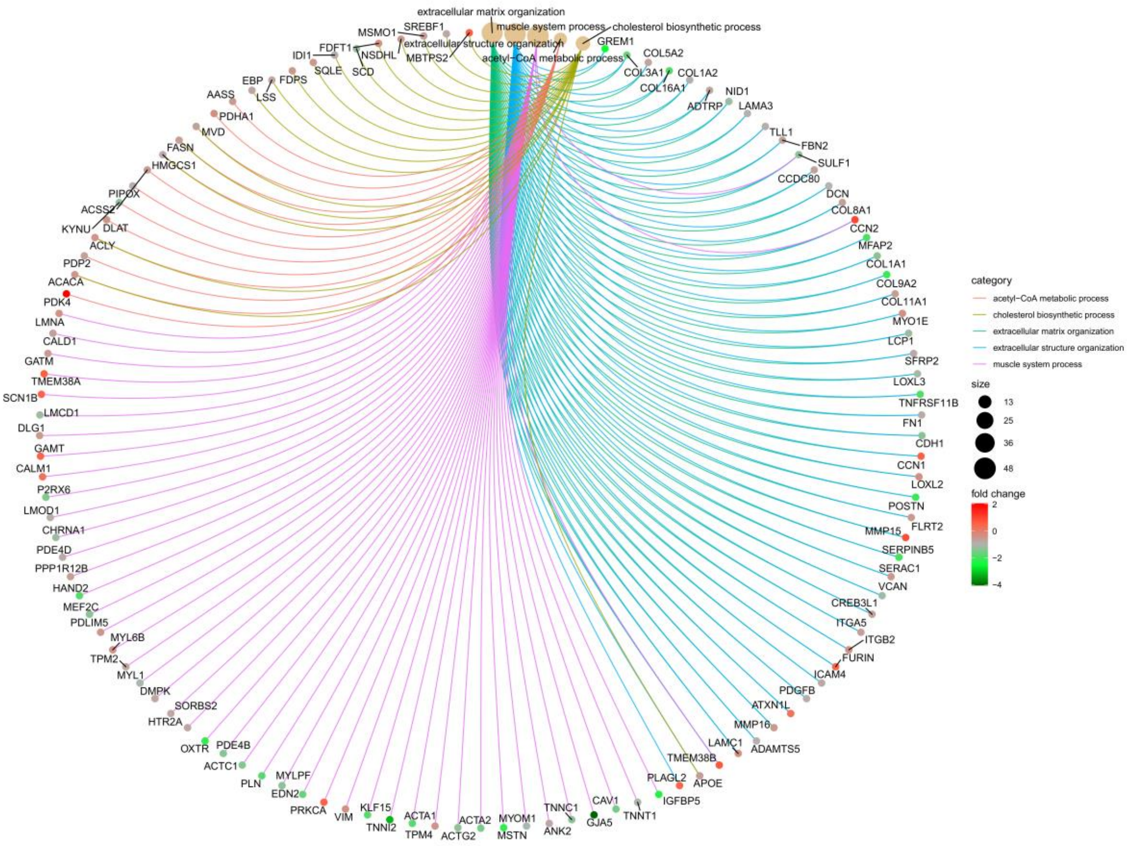

Supplementary Figure 4. Circular plot representing main pathways altered by 10 μM SA treatment in mRPE cells. Proteins in red are expressed at higher levels while proteins in green are expressed at lower levels than controls.

Table S1. DEGs in mRPE cells after SA treatment.

Ensembl ID	GeneID	log2FoldChange	padj	Gene Description
ENSMMUG00000001810	GREM1	−2.48	5.58E−51	gremlin 1, DAN family BMP antagonist
ENSMMUG00000041480	IGFBP5	−2.31	1.62E−50	insulin like growth factor binding protein 5
ENSMMUG00000021286	COL3A1	−1.66	2.74E−21	collagen type III alpha 1 chain
ENSMMUG00000021290	COL5A2	−0.73	3.23E−20	collagen type V alpha 2 chain
ENSMMUG00000000902	PLA1A	−1.51	3.72E−20	phospholipase A1 member A
ENSMMUG00000021267	KCNN4	−2.09	4.77E−17	potassium calcium−activated channel subfamily N member 4
ENSMMUG00000010393	CDH3	−2.29	3.50E−15	cadherin 3
ENSMMUG00000011170	HDAC9	−2.02	1.84E−14	histone deacetylase 9
ENSMMUG00000000698	ANGPTL4	4.58	1.11E−13	angiopoietin like 4
ENSMMUG00000006169	TNNT1	−1.07	1.80E−13	troponin T1, slow skeletal type
ENSMMUG00000013446	ANXA8	−1.47	3.04E−13	annexin A8
ENSMMUG00000014122	CAV1	−1.52	5.87E−13	caveolin 1
ENSMMUG00000001780	CPT1A	0.97	1.07E−12	carnitine palmitoyltransferase 1A
ENSMMUG00000020987	FABP3	−1.50	1.07E−12	fatty acid binding protein 4
ENSMMUG00000023138	BRD2	0.57	2.52E−12	bromodomain containing 2
ENSMMUG00000007087	SLC22A5	0.85	6.83E−12	solute carrier family 22 member 5
ENSMMUG00000013507	LAD1	−2.42	1.15E−11	ladinin 1
ENSMMUG00000008055	SMYD1	−3.59	1.23E−11	SET and MYND domain containing 1
ENSMMUG00000014420	RLBP1	−1.11	4.04E−11	retinaldehyde binding protein 1
ENSMMUG00000048526	CLIP2	0.87	1.99E−10	CAP−Gly domain containing linker protein 2
ENSMMUG00000022581	KCNMB1	−1.60	1.99E−10	#N/A
ENSMMUG00000017215	TM4SF1	−1.23	2.22E−10	#N/A
ENSMMUG00000016104	CSPG4	−1.27	2.55E−10	chondroitin sulfate proteoglycan 4
ENSMMUG00000023398	COL16A1	−1.82	2.78E−10	collagen type XVI alpha 1 chain
ENSMMUG00000001803	GJA5	−4.06	1.00E−09	gap junction protein alpha 5
ENSMMUG00000003610	SORCS3	−1.11	1.02E−09	sortilin related VPS10 domain containing receptor 3
ENSMMUG00000021931	AOX1	−0.73	1.99E−09	aldehyde oxidase 1
ENSMMUG00000013798	FADS1	−1.25	3.38E−09	fatty acid desaturase 1
ENSMMUG00000014984	KRT4	−2.43	3.38E−09	keratin, type II cytoskeletal 4
ENSMMUG00000004233	LRIG3	−0.87	3.42E−09	leucine rich repeats and immunoglobulin like domains 3
ENSMMUG00000008610	OSBPL10	−0.54	3.42E−09	oxysterol binding protein like 10
ENSMMUG00000018488	HSPB3	−1.94	5.65E−09	heat shock protein family B (small) member 3
ENSMMUG00000007338	TXNIP	0.70	7.94E−09	thioredoxin interacting protein
ENSMMUG00000045591		−25.86	1.94E−08	#N/A
ENSMMUG00000014782	STC2	−1.23	1.96E−08	stanniocalcin 2

ENSMMUG00000021389	PDK4	2.07	2.88E−08	pyruvate dehydrogenase kinase 4
ENSMMUG00000006955	TMEM151A	−27.48	3.35E−08	transmembrane protein 151A
ENSMMUG00000044640	MYEOV	−1.65	5.13E−08	
ENSMMUG00000013297	CAVIN3	−0.92	6.57E−08	#N/A
ENSMMUG00000040385	UBD	−1.08	6.61E−08	#N/A
ENSMMUG00000044432	RASSF3	−0.70	6.80E−08	#N/A
ENSMMUG00000000623	P3H2	0.55	7.13E−08	prolyl 3−hydroxylase 2
ENSMMUG00000005631	NOV	−1.74	8.74E−08	cellular communication network factor 3
ENSMMUG00000048768		−1.36	8.96E−08	#N/A
ENSMMUG00000048823	STATH	−1.18	1.59E−07	
ENSMMUG00000002038	CEMIP	−1.28	1.67E−07	cell migration inducing hyaluronidase 1
ENSMMUG00000013831	ASRGL1	0.93	2.41E−07	asparaginase and isoaspartyl peptidase 1
ENSMMUG00000039654	COL1A2	−0.96	2.61E−07	collagen type I alpha 2 chain
ENSMMUG00000018653	KRT19	−1.87	2.63E−07	keratin, type I cytoskeletal 19
ENSMMUG00000015115	FAM46B	−2.88	2.70E−07	terminal nucleotidyltransferase 5B
ENSMMUG00000018265	PRR5L	−1.11	2.91E−07	proline rich 5 like
ENSMMUG00000021329	NAV3	−0.73	3.38E−07	neuron navigator 3
ENSMMUG00000044056	EBP	−0.75	4.75E−07	#N/A
ENSMMUG00000004347	ADTRP	−0.76	4.97E−07	androgen dependent TFPI regulating protein
ENSMMUG00000015051	PDE2A	−1.09	5.25E−07	#N/A
ENSMMUG00000018815	KLF10	0.70	5.48E−07	Kruppel like factor 10
ENSMMUG00000012716	NID1	−1.20	5.69E−07	nidogen 1
ENSMMUG00000004330	PAMR1	−1.18	5.69E−07	peptidase domain containing associated with muscle regeneration 1
ENSMMUG00000030384	RASA4	−0.80	5.69E−07	RAS p21 protein activator 4B
ENSMMUG00000013067	EMP3	−0.76	6.93E−07	epithelial membrane protein 3
ENSMMUG00000011377	EPB41	−0.52	7.37E−07	erythrocyte membrane protein band 4.1
ENSMMUG00000028743	FKRP	0.70	8.30E−07	fukutin related protein
ENSMMUG00000004321	LAMA3	−0.98	8.32E−07	laminin subunit alpha 3
ENSMMUG00000044910		−0.90	1.08E−06	#N/A
ENSMMUG00000005618	TNNC1	−1.31	1.31E−06	troponin C1, slow skeletal and cardiac type
ENSMMUG00000013786	DSTN	−0.53	1.33E−06	destrin, actin depolymerizing factor
ENSMMUG00000006265	NES	−1.06	1.33E−06	nestin
ENSMMUG00000022170	PFKFB2	−0.86	1.33E−06	6−phosphofructo−2−kinase/fructose−2,6−biphosphatase 2
ENSMMUG00000000613	RAB11FIP3	0.65	1.33E−06	RAB11 family interacting protein 3
ENSMMUG00000013288		−1.27	1.73E−06	#N/A
ENSMMUG00000007143	WRNIP1	0.54	1.73E−06	WRN helicase interacting protein 1
ENSMMUG00000008334	GPR1	−1.01	1.76E−06	G protein−coupled receptor 1
ENSMMUG00000015575	CAGE1	−21.51	2.29E−06	cancer antigen 1

ENSMMUG00000000327	FADS2	−1.10	2.29E−06	fatty acid desaturase 2
ENSMMUG00000001616	PADI2	−3.32	2.29E−06	peptidyl arginine deiminase 2
ENSMMUG00000018925	TGM2	−1.25	2.35E−06	transglutaminase 2
ENSMMUG00000008633	ANK2	−0.77	2.69E−06	ankyrin 2
ENSMMUG00000013764	IGFBP3	−0.93	2.69E−06	insulin like growth factor binding protein 3
ENSMMUG00000021586	TRIM55	−1.21	2.69E−06	tripartite motif containing 55
ENSMMUG00000000311	JSRP1	−0.84	2.80E−06	junctional sarcoplasmic reticulum protein 1
ENSMMUG00000007069	MARCH11	−24.23	2.80E−06	membrane associated ring−CH−type finger 11
ENSMMUG00000003893		−23.95	3.10E−06	#N/A
ENSMMUG00000012161	ANGPTL2	−0.76	3.10E−06	angiopoietin like 2
ENSMMUG00000015799	TLL1	−0.89	3.13E−06	tolloid like 1
ENSMMUG00000003370	NFATC4	−2.04	3.29E−06	nuclear factor of activated T cells 4
ENSMMUG00000047195	KCNQ1OT1_3	−22.57	3.31E−06	#N/A
ENSMMUG00000019892	MYOM1	−1.07	3.46E−06	myomesin 1
ENSMMUG00000020392	SMS	−0.63	3.54E−06	spermine synthase
ENSMMUG00000010682	FBN2	−0.66	3.75E−06	fibrillin 2
ENSMMUG00000037887	RBMXL1	−0.66	3.85E−06	RBMX like 1
ENSMMUG00000022329	LSS	−0.77	3.89E−06	lanosterol synthase
ENSMMUG00000042385		−22.58	4.16E−06	#N/A
ENSMMUG00000006734	TIMP3	−1.03	4.32E−06	#N/A
ENSMMUG00000015418	SLC25A20	0.82	4.61E−06	solute carrier family 25 member 20
ENSMMUG00000023144	FDPS	−0.51	4.83E−06	farnesyl diphosphate synthase
ENSMMUG00000023274	SULF1	−1.24	4.83E−06	sulfatase 1
ENSMMUG00000008924	PLLP	−1.69	4.99E−06	plasmolipin
ENSMMUG00000002429	CAT	0.70	5.71E−06	catalase
ENSMMUG00000004837	SYT8	−1.49	5.87E−06	synaptotagmin 8
ENSMMUG00000015971	SORD	−0.62	6.06E−06	sorbitol dehydrogenase
ENSMMUG00000003061	CCDC80	−0.71	6.40E−06	coiled−coil domain containing 80
ENSMMUG00000022369	MRPS28	−0.60	6.52E−06	tumor protein D52
ENSMMUG00000039944	RASGRF2	−0.57	6.58E−06	Ras protein specific guanine nucleotide releasing factor 2
ENSMMUG00000033817		−23.36	6.75E−06	#N/A
ENSMMUG00000014307	MSTN	−2.18	6.81E−06	myostatin
ENSMMUG00000015614	TRPV2	−1.35	6.84E−06	transient receptor potential cation channel subfamily V member 2
ENSMMUG00000006855	ACOT2	−1.26	6.85E−06	acyl−CoA thioesterase 2
ENSMMUG00000009349	ACACA	−0.47	6.86E−06	acetyl−CoA carboxylase alpha
ENSMMUG00000004439		−0.48	6.90E−06	#N/A
ENSMMUG00000015179	PAWR	−0.59	7.69E−06	pro−apoptotic WT1 regulator
ENSMMUG00000044197		−1.50	9.02E−06	#N/A

ENSMMUG00000002700	SLC47A1	0.99	9.02E−06	solute carrier family 47 member 1
ENSMMUG00000008855	TRABD2A	−1.51	9.02E−06	TraB domain containing 2A
ENSMMUG00000016997	DCN	−1.03	9.13E−06	decorin
ENSMMUG00000012874	PGK1	−0.51	9.13E−06	phosphoglycerate kinase 1
ENSMMUG00000000567	BAMBI	0.89	9.46E−06	#N/A
ENSMMUG00000009341	ACTA2	−1.51	9.82E−06	actin alpha 2, smooth muscle
ENSMMUG00000033718	mml−mir−1262	−22.76	1.12E−05	mml−mir−1262
ENSMMUG00000046346		−22.85	1.17E−05	#N/A
ENSMMUG00000002605	UCP2	−0.90	1.30E−05	uncoupling protein 2
ENSMMUG00000003535	SQLE	−0.50	1.41E−05	squalene epoxidase
ENSMMUG00000021252	TRIP6	−0.69	1.45E−05	thyroid hormone receptor interactor 6
ENSMMUG00000022895	DNAJB5	−0.86	1.51E−05	DnaJ heat shock protein family (Hsp40) member B5
ENSMMUG00000000729		−0.46	1.55E−05	complement factor B
ENSMMUG00000015503	ZFYVE27	0.51	1.56E−05	zinc finger FYVE−type containing 27
ENSMMUG00000040660	HDGF	−0.57	1.59E−05	heparin binding growth factor
ENSMMUG00000010538	ACADVL	0.58	1.63E−05	acyl−CoA dehydrogenase very long chain
ENSMMUG00000001547	ZNF106	−0.73	1.69E−05	zinc finger protein 106
ENSMMUG00000012406	PCOLCE2	−0.48	1.86E−05	procollagen C−endopeptidase enhancer 2
ENSMMUG00000045697	Y_RNA	−22.26	1.95E−05	#N/A
ENSMMUG00000018487	KLHL30	−1.63	2.04E−05	kelch like family member 30
ENSMMUG00000017049	TSPAN13	0.69	2.13E−05	tetraspanin 13
ENSMMUG00000018349		−0.53	2.25E−05	#N/A
ENSMMUG00000014699	COL7A1	−1.04	2.25E−05	
ENSMMUG00000004998	DLGAP3	−2.32	2.25E−05	DLG associated protein 3
ENSMMUG00000017115	IDI1	−0.86	2.25E−05	isopentenyl−diphosphate delta isomerase 1
ENSMMUG00000010847	DKK1	−0.75	2.99E−05	dickkopf WNT signaling pathway inhibitor 1
ENSMMUG00000022478	FGFR1	0.53	2.99E−05	fibroblast growth factor receptor 1
ENSMMUG00000010817	SCD	−1.18	3.18E−05	Stearoyl−CoA Desaturase
ENSMMUG00000022439	TAS2R4	−21.82	3.19E−05	taste 2 receptor member 4
ENSMMUG00000048037	Y_RNA	−21.98	3.19E−05	#N/A
ENSMMUG00000002935	COL8A1	−0.62	3.38E−05	collagen type VIII alpha 1 chain
ENSMMUG00000002779	CHST2	1.14	3.64E−05	carbohydrate sulfotransferase 2
ENSMMUG00000011160	POLG	0.46	3.64E−05	DNA polymerase gamma, catalytic subunit
ENSMMUG00000018918	SCG2	−1.77	3.89E−05	secretogranin II
ENSMMUG00000049206		−0.40	4.38E−05	#N/A
ENSMMUG00000017683	ACTG2	−1.34	4.38E−05	actin gamma 2, smooth muscle
ENSMMUG00000014150	CTGF	1.01	4.49E−05	cellular communication network factor 2
ENSMMUG00000022218	ANXA3	−0.82	4.62E−05	annexin A3

ENSMMUG00000033545	mml−mir−619	−21.51	4.62E−05	#N/A
ENSMMUG00000009584	NEO1	0.86	4.62E−05	neogenin 1
ENSMMUG00000029123		1.33	4.80E−05	#N/A
ENSMMUG00000048901		−0.92	5.02E−05	#N/A
ENSMMUG00000004692	CHAT	−2.43	5.02E−05	choline O−acetyltransferase
ENSMMUG00000045554	CAV2	−0.68	5.24E−05	#N/A
ENSMMUG00000037491	U6	−21.33	5.24E−05	#N/A
ENSMMUG00000003500	TCEAL1	−0.73	5.28E−05	transcription elongation factor A like 1
ENSMMUG00000009274	CLDN11	−21.33	5.34E−05	#N/A
ENSMMUG00000011253	ADH7	−1.03	5.35E−05	#N/A
ENSMMUG00000016102	KCNH1	−0.99	5.87E−05	potassium voltage−gated channel subfamily H member 1
ENSMMUG00000047454	SYNC	−0.71	6.70E−05	syncoilin, intermediate filament protein
ENSMMUG00000009674	ELANE	−21.08	7.20E−05	elastase, neutrophil expressed
ENSMMUG00000029263	PKD1	0.65	7.32E−05	polycystin 1, transient receptor potential channel interacting
ENSMMUG00000001420	MFAP2	−1.94	7.37E−05	#N/A
ENSMMUG00000001328	ALDH3A1	−1.06	7.97E−05	aldehyde dehydrogenase 3 family member A1
ENSMMUG00000021724	NUP214	0.45	7.97E−05	nucleoporin 214
ENSMMUG00000046424	C12orf75	−0.57	8.11E−05	chromosome 11 C12orf75 homolog
ENSMMUG00000009638	GLIPR1	−0.78	8.17E−05	#N/A
ENSMMUG00000022961	SLC11A2	−0.39	8.30E−05	solute carrier family 11 member 2
ENSMMUG00000031534	ANKUB1	−20.90	8.47E−05	ankyrin repeat and ubiquitin domain containing 1
ENSMMUG00000012889	PCYT2	−0.61	9.09E−05	phosphate cytidylyltransferase 2, ethanolamine
ENSMMUG00000003135	TPD52L1	−1.15	9.36E−05	#N/A
ENSMMUG00000016461	EFR3B	1.31	9.54E−05	EFR3 homolog B
ENSMMUG00000008341	ARHGAP24	−0.82	9.74E−05	Rho GTPase activating protein 24
ENSMMUG00000001467	COL1A1	−1.37	9.76E−05	collagen type I alpha 1 chain
ENSMMUG00000000040	EGLN3	−1.15	9.76E−05	egl−9 family hypoxia inducible factor 3
ENSMMUG00000010979	S100A4	−0.75	9.91E−05	S100 calcium binding protein A4
ENSMMUG00000009312	LTA4H	0.38	9.95E−05	leukotriene A4 hydrolase
ENSMMUG00000014149	LYN	0.71	1.07E−04	LYN proto−oncogene, Src family tyrosine kinase
ENSMMUG00000031129	LRRN4CL	−1.49	1.11E−04	LRRN4 C−terminal like
ENSMMUG00000018664	COL9A2	−2.11	1.27E−04	collagen type IX alpha 2 chain
ENSMMUG00000001245	ZNF532	−0.38	1.32E−04	zinc finger protein 532
ENSMMUG00000048520		−20.44	1.34E−04	#N/A
ENSMMUG00000043332	LAT	−1.07	1.34E−04	linker for activation of T cells
ENSMMUG00000003674	TREM1	−1.03	1.35E−04	triggering receptor expressed on myeloid cells 1
ENSMMUG00000000108	NCKAP1L	−2.15	1.43E−04	NCK associated protein 1 like
ENSMMUG00000004103	COL11A1	−0.63	1.45E−04	collagen type XI alpha 1 chain

ENSMMUG00000010551	CLDN7	−17.26	1.46E−04	claudin 7
ENSMMUG00000016726	PDP2	−0.62	1.47E−04	pyruvate dehyrogenase phosphatase catalytic subunit 2
ENSMMUG00000012933	MYO1E	−0.32	1.54E−04	myosin IE
ENSMMUG00000001141	BNC2	−0.53	1.57E−04	basonuclin 2
ENSMMUG00000018268	RAG2	−2.75	1.61E−04	recombination activating 2
ENSMMUG00000017033	LCP1	−1.20	1.63E−04	lymphocyte cytosolic protein 1
ENSMMUG00000022903	INPP4A	0.55	1.63E−04	inositol polyphosphate−4−phosphatase type I A
ENSMMUG00000008329	CHN2	−1.24	1.64E−04	chimerin 2
ENSMMUG00000023620	POLR2A	0.60	1.64E−04	RNA polymerase II subunit A
ENSMMUG00000015868	RDH11	−0.40	1.64E−04	retinol dehydrogenase 11
ENSMMUG00000000896	TGFB1I1	−0.85	1.64E−04	transforming growth factor beta 1 induced transcript 1
ENSMMUG00000019085	BDH2	−0.59	1.68E−04	3−hydroxybutyrate dehydrogenase 2
ENSMMUG00000015569	ABCC10	0.54	1.74E−04	ATP binding cassette subfamily C member 10
ENSMMUG00000023081	SCHIP1	−0.78	1.78E−04	schwannomin interacting protein 1
ENSMMUG00000043691	PPP1R3B	−0.65	1.85E−04	#N/A
ENSMMUG00000015851	SFRP2	−0.83	1.85E−04	secreted frizzled related protein 2
ENSMMUG00000020694	RAB27A	−0.97	1.94E−04	RAB27A, member RAS oncogene family
ENSMMUG00000046062	DOK7	−1.19	1.97E−04	docking protein 7
ENSMMUG00000014520	ITIH1	−2.38	2.00E−04	inter−alpha−trypsin inhibitor heavy chain 1
ENSMMUG00000047299	SLC18A3	−2.01	2.00E−04	solute carrier family 18 member A3
ENSMMUG00000031427	TRAF5	−0.54	2.00E−04	TNF receptor associated factor 5
ENSMMUG00000047012		−20.12	2.07E−04	#N/A
ENSMMUG00000003734	SLC10A6	−20.06	2.09E−04	solute carrier family 10 member 6
ENSMMUG00000039700	HIST2H2AA3	0.69	2.10E−04	#N/A
ENSMMUG00000031161	CHML	−1.27	2.19E−04	#N/A
ENSMMUG00000021559	JCAD	1.14	2.19E−04	junctional cadherin 5 associated
ENSMMUG00000004771	CXCL14	−0.94	2.24E−04	C−X−C motif chemokine ligand 14
ENSMMUG00000015783	TPM4	−0.43	2.37E−04	tropomyosin 4
ENSMMUG00000022221	CHD3	−0.55	2.43E−04	chromodomain helicase DNA binding protein 3
ENSMMUG00000012452	TGM1	−1.06	2.57E−04	transglutaminase 1
ENSMMUG00000002138	LOXL3	−1.13	2.64E−04	lysyl oxidase like 3
ENSMMUG00000007593	PHKA1	0.65	2.64E−04	phosphorylase kinase regulatory subunit alpha 1
ENSMMUG00000030786	TRPC4	−1.19	2.64E−04	transient receptor potential cation channel subfamily C member 4
ENSMMUG00000034946	Y_RNA	−19.83	2.68E−04	#N/A
ENSMMUG00000014176	CYTIP	−1.56	2.73E−04	cytohesin 1 interacting protein
ENSMMUG00000018386	SH3RF2	−0.82	2.78E−04	SH3 domain containing ring finger 2
ENSMMUG00000000671	IDS	0.48	2.83E−04	iduronate 2−sulfatase
ENSMMUG00000005669	OPN3	−0.73	2.83E−04	opsin 3

ENSMMUG00000022112	DPPA2	−0.86	2.87E−04	developmental pluripotency associated 2
ENSMMUG00000016485	TNFRSF11B	−1.89	2.87E−04	TNF receptor superfamily member 11b
ENSMMUG00000019751	PPFIBP2	−0.66	2.95E−04	PPFIA binding protein 2
ENSMMUG00000020956	PARVA	−0.33	2.97E−04	parvin alpha
ENSMMUG00000037043	U5	−19.63	3.04E−04	U5 spliceosomal RNA
ENSMMUG00000013099	PAPPA	−1.33	3.24E−04	pappalysin 1
ENSMMUG00000006262	ABL2	−0.49	3.24E−04	ABL proto−oncogene 2, non−receptor tyrosine kinase
ENSMMUG00000040249	PXT1	−19.58	3.25E−04	#N/A
ENSMMUG00000000242	PPP1R3C	−0.51	3.54E−04	protein phosphatase 1 regulatory subunit 3C
ENSMMUG00000004019	IZUMO1	−19.47	3.76E−04	izumo sperm−egg fusion 1
ENSMMUG00000017963	MREG	−0.67	3.85E−04	#N/A
ENSMMUG00000015775	MGEA5	0.65	3.87E−04	O−GlcNAcase
ENSMMUG00000047499	VSIG10	0.69	3.95E−04	V−set and immunoglobulin domain containing 10
ENSMMUG00000019481	SLC26A7	−1.36	4.12E−04	solute carrier family 26 member 7
ENSMMUG00000034752	U6	19.24	4.23E−04	U6 spliceosomal RNA
ENSMMUG00000005515	ABCC5	0.55	4.25E−04	ATP binding cassette subfamily C member 5
ENSMMUG00000019268	CD82	−0.70	4.25E−04	CD82 molecule
ENSMMUG00000019111	TRIM29	−0.93	4.25E−04	tripartite motif containing 29
ENSMMUG00000012321	FN1	−0.86	4.27E−04	fibronectin 1
ENSMMUG00000018663	EEFSEC	0.67	4.34E−04	eukaryotic elongation factor, selenocysteine−tRNA specific
ENSMMUG00000011850	CDH1	−1.38	4.37E−04	cadherin 1
ENSMMUG00000015407	PDLIM3	−1.00	4.37E−04	PDZ and LIM domain 3
ENSMMUG00000008847	SH2D5	−1.03	4.37E−04	SH2 domain containing 5
ENSMMUG00000022086	ACTA1	−1.80	4.52E−04	actin alpha 1, skeletal muscle
ENSMMUG00000013197	CYR61	0.58	4.57E−04	cellular communication network factor 1
ENSMMUG00000016711	H6PD	0.52	4.80E−04	hexose−6−phosphate dehydrogenase/glucose 1−dehydrogenase
ENSMMUG00000009738	SLC25A29	−0.73	4.81E−04	solute carrier family 25 member 29
ENSMMUG00000027221	mml−mir−593	−19.11	4.90E−04	mml−mir−593
ENSMMUG00000039240		18.96	5.40E−04	#N/A
ENSMMUG00000002163		−0.69	5.40E−04	#N/A
ENSMMUG00000015101	SHISA8	−19.07	5.40E−04	shisa family member 8
ENSMMUG00000017222	DGKQ	0.60	5.63E−04	diacylglycerol kinase theta
ENSMMUG00000003749	NT5C2	0.41	6.16E−04	5'−nucleotidase, cytosolic II
ENSMMUG00000014646	NTF3	−0.48	6.16E−04	neurotrophin 3
ENSMMUG00000020283	EIF3H	−0.34	6.21E−04	eukaryotic translation initiation factor 3 subunit H
ENSMMUG00000037580	HSD17B2	−0.80	6.34E−04	hydroxysteroid 17−beta dehydrogenase 2
ENSMMUG00000022227	ZNF445	0.44	6.34E−04	zinc finger protein 445
ENSMMUG00000013098	AKR1B10	0.62	6.44E−04	aldo−keto reductase family 1 member B10

ENSMMUG00000014024	DCHS2	−1.09	6.46E−04	dachsous cadherin−related 2
ENSMMUG00000022771	PASD1	−18.87	6.46E−04	PAS domain containing repressor 1
ENSMMUG00000019925	DGCR2	0.49	6.56E−04	DiGeorge syndrome critical region gene 2
ENSMMUG00000007542	TNFRSF10A	−0.52	6.56E−04	TNF receptor superfamily member 10a
ENSMMUG00000017745	ANGPT1	−2.10	6.66E−04	angiopoietin 1
ENSMMUG00000010115	CHST15	0.56	6.66E−04	carbohydrate sulfotransferase 15
ENSMMUG00000019357	ELP1	0.43	6.80E−04	elongator complex protein 1
ENSMMUG00000017954	ADGRF2	−2.97	6.97E−04	#N/A
ENSMMUG00000019891	AKR1C8P	−1.05	7.06E−04	aldo−keto reductase family 1 member C15
ENSMMUG00000012039	CYB5B	−0.34	7.18E−04	cytochrome b5 type B (outer mitochondrial membrane)
ENSMMUG00000037990		−18.68	7.27E−04	#N/A
ENSMMUG00000012400	ASB2	−1.01	7.32E−04	ankyrin repeat and SOCS box containing 2
ENSMMUG00000002054	KLF11	0.44	7.32E−04	Kruppel like factor 11
ENSMMUG00000001742	TGFBR3	−0.70	7.36E−04	transforming growth factor beta receptor 3
ENSMMUG00000004838	TNNI2	−3.28	7.49E−04	troponin I2, fast skeletal type
ENSMMUG00000003721	KLF15	−1.83	7.54E−04	Kruppel like factor 15
ENSMMUG00000037604		−18.63	7.63E−04	#N/A
ENSMMUG00000010002	KRT80	−0.85	7.81E−04	keratin 80
ENSMMUG00000008872	INA	0.47	7.92E−04	internexin neuronal intermediate filament protein alpha
ENSMMUG00000009160	UBE2Q2	−0.42	7.98E−04	ubiquitin conjugating enzyme E2 Q2
ENSMMUG00000007814	GPM6A	−0.61	8.16E−04	glycoprotein M6A
ENSMMUG00000017567	AHSG	−18.52	8.31E−04	alpha 2−HS glycoprotein
ENSMMUG00000010453	FOLR2	−18.52	8.31E−04	folate receptor beta
ENSMMUG00000016804	NUDT4	−0.42	8.58E−04	#N/A
ENSMMUG00000008749	ARHGEF15	−18.48	8.59E−04	Rho guanine nucleotide exchange factor 15
ENSMMUG00000006546	ATP6V0A2	0.43	8.59E−04	ATPase H+ transporting V0 subunit a2
ENSMMUG00000005646	DGKD	0.56	8.59E−04	diacylglycerol kinase delta
ENSMMUG00000037596	U6	−18.44	8.99E−04	U6 spliceosomal RNA
ENSMMUG00000015325	COL17A1	−2.91	9.06E−04	collagen type XVII alpha 1 chain
ENSMMUG00000017131	XYLT1	18.36	9.06E−04	xylosyltransferase 1
ENSMMUG00000015689	ADGRG1	−1.24	9.12E−04	adhesion G protein−coupled receptor G1
ENSMMUG00000041050		18.30	9.25E−04	#N/A
ENSMMUG00000009289	CBS	−1.13	9.25E−04	cystathionine beta−synthase
ENSMMUG00000040765		18.34	9.31E−04	#N/A
ENSMMUG00000014167	ESRRG	−18.35	9.58E−04	estrogen related receptor gamma
ENSMMUG00000021516	CLU	0.46	9.85E−04	clusterin
ENSMMUG00000012864	PDIA6	−0.39	9.85E−04	protein disulfide isomerase family A member 6
ENSMMUG00000018378	PRKCA	0.49	9.99E−04	protein kinase C alpha

ENSMMUG00000003193	VIM	−0.34	9.99E−04	vimentin
ENSMMUG00000002264	LPO	−18.32	1.01E−03	lactoperoxidase
ENSMMUG00000041799	DYNLL2	0.46	1.01E−03	dynein light chain LC8−type 2
ENSMMUG00000016214	EDN2	−1.67	1.01E−03	endothelin 2
ENSMMUG00000021847	MYLPF	−1.31	1.05E−03	myosin light chain, phosphorylatable, fast skeletal muscle
ENSMMUG00000018341	PLCH1	−0.80	1.05E−03	phospholipase C eta 1
ENSMMUG00000030590	C10orf128	−18.12	1.11E−03	transmembrane protein 273
ENSMMUG00000004598	BPGM	−0.51	1.11E−03	bisphosphoglycerate mutase
ENSMMUG00000019211	FDFT1	−0.31	1.14E−03	#N/A
ENSMMUG00000000261	ADORA2A	−1.07	1.15E−03	#N/A
ENSMMUG00000031026	CACNG2	18.05	1.19E−03	calcium voltage−gated channel auxiliary subunit gamma 2
ENSMMUG00000045439	PLN	−1.79	1.21E−03	#N/A
ENSMMUG00000015966	ADH6	18.06	1.21E−03	alcohol dehydrogenase 6 (class V)
ENSMMUG00000042362	CLEC2B	−0.99	1.21E−03	#N/A
ENSMMUG00000001522	ASS1	−0.61	1.24E−03	argininosuccinate synthase 1
ENSMMUG00000018931	ACLY	−0.42	1.25E−03	ATP citrate lyase
ENSMMUG00000015330	CCDC50	−0.45	1.26E−03	coiled−coil domain containing 50
ENSMMUG00000001609	OSMR	0.77	1.26E−03	oncostatin M receptor
ENSMMUG00000047171		−18.02	1.27E−03	#N/A
ENSMMUG00000002456	MAPK8IP1	0.58	1.28E−03	mitogen−activated protein kinase 8 interacting protein 1
ENSMMUG00000009001	TMEM131L	0.44	1.30E−03	transmembrane 131 like
ENSMMUG00000032243	FBLIM1	−0.72	1.39E−03	filamin binding LIM protein 1
ENSMMUG00000010238	LGALSL	−0.61	1.45E−03	#N/A
ENSMMUG00000000492	HSPA2	−1.01	1.48E−03	#N/A
ENSMMUG00000037684	SIRPB1	−1.22	1.51E−03	signal−regulatory protein beta−1
ENSMMUG00000014347	B3GNT8	−17.90	1.53E−03	UDP−GlcNAc:betaGal beta−1,3−N−acetylglucosaminyltransferase 8
ENSMMUG00000031282	TSPAN4	−0.53	1.54E−03	tetraspanin 4
ENSMMUG00000016658	ENTPD4	−0.30	1.57E−03	ectonucleoside triphosphate diphosphohydrolase 4
ENSMMUG00000017568	DLAT	−0.44	1.57E−03	dihydrolipoamide S−acetyltransferase
ENSMMUG00000006659	ALDH1A3	−0.43	1.62E−03	aldehyde dehydrogenase 1 family member A3
ENSMMUG00000035721		−17.83	1.62E−03	#N/A
ENSMMUG00000039139	RIMS3	−0.67	1.64E−03	regulating synaptic membrane exocytosis 3
ENSMMUG00000009842	FGF7	−1.55	1.69E−03	fibroblast growth factor 7
ENSMMUG00000012307	POSTN	−2.11	1.69E−03	periostin
ENSMMUG00000009475	LYPD6B	−0.74	1.70E−03	LY6/PLAUR domain containing 6B
ENSMMUG00000002825	KCNF1	17.63	1.72E−03	#N/A
ENSMMUG00000044620		17.56	1.86E−03	#N/A
ENSMMUG00000023518	KCNH5	−0.36	1.99E−03	potassium voltage−gated channel subfamily H member 5

ENSMMUG00000038101		17.49	2.02E−03	#N/A
ENSMMUG00000010104	GATAD1	0.44	2.02E−03	GATA zinc finger domain containing 1
ENSMMUG00000013343	MPRIP	0.41	2.02E−03	myosin phosphatase Rho interacting protein
ENSMMUG00000014181	B3GNT2	−0.49	2.06E−03	UDP−GlcNAc:betaGal beta−1,3−N−acetylglucosaminyltransferase 2
ENSMMUG00000010743	CHRNA5	1.63	2.07E−03	cholinergic receptor nicotinic alpha 5 subunit
ENSMMUG00000004552	DDT	−0.44	2.07E−03	D−dopachrome tautomerase
ENSMMUG00000011588	MYO9B	0.46	2.10E−03	
ENSMMUG00000010113	SRD5A3	0.64	2.15E−03	steroid 5 alpha−reductase 3
ENSMMUG00000019156	PARD3	−0.32	2.21E−03	par−3 family cell polarity regulator
ENSMMUG00000017490	C10orf10	−1.31	2.21E−03	#N/A
ENSMMUG00000001633	GSDMD	−0.59	2.21E−03	gasdermin D
ENSMMUG00000013540	RANGRF	−1.00	2.21E−03	RAN guanine nucleotide release factor
ENSMMUG00000016757	STRADA	0.42	2.21E−03	STE20 related adaptor alpha
ENSMMUG00000047833		−0.33	2.22E−03	#N/A
ENSMMUG00000003595	PEX5L	17.32	2.29E−03	peroxisomal biogenesis factor 5 like
ENSMMUG00000002021	BDH1	−0.66	2.31E−03	3−hydroxybutyrate dehydrogenase 1
ENSMMUG00000020942	LGR5	−1.78	2.31E−03	leucine rich repeat containing G protein−coupled receptor 5
ENSMMUG00000026873	mml−mir−378a	17.29	2.31E−03	microRNA 378a
ENSMMUG00000017187	LGALS1	−0.48	2.32E−03	#N/A
ENSMMUG00000007027	ACTC1	−1.41	2.33E−03	actin alpha cardiac muscle 1
ENSMMUG00000019082	SFRP1	−0.50	2.34E−03	secreted frizzled related protein 1
ENSMMUG00000012776	DHH	17.29	2.36E−03	desert hedgehog signaling molecule
ENSMMUG00000007490	PDE4B	−1.36	2.36E−03	phosphodiesterase 4B
ENSMMUG00000019298	SMCO3	−17.30	2.37E−03	#N/A
ENSMMUG00000047999		16.57	2.37E−03	#N/A
ENSMMUG00000008886	CRTC3	0.43	2.37E−03	CREB regulated transcription coactivator 3
ENSMMUG00000024417	U6	17.20	2.38E−03	U6 spliceosomal RNA
ENSMMUG00000009703	OXTR	−2.16	2.43E−03	oxytocin receptor
ENSMMUG00000003310	TTC7A	0.49	2.46E−03	tetratricopeptide repeat domain 7A
ENSMMUG00000047533		−1.51	2.52E−03	
ENSMMUG00000003896	ARHGAP6	−0.61	2.57E−03	Rho GTPase activating protein 6
ENSMMUG00000000792	FAM134A	0.39	2.60E−03	reticulophagy regulator family member 2
ENSMMUG00000010807	C1orf189	−17.25	2.62E−03	#N/A
ENSMMUG00000009499	MEST	−0.75	2.65E−03	mesoderm specific transcript
ENSMMUG00000019967	KLHL1	−17.16	2.65E−03	kelch like family member 1
ENSMMUG00000037083	NRON	−17.19	2.77E−03	#N/A
ENSMMUG00000001043	PRUNE2	−0.68	2.77E−03	prune homolog 2 with BCH domain
ENSMMUG00000046668		−0.88	2.92E−03	#N/A

ENSMMUG00000004009	OPLAH	−0.69	2.92E−03	5−oxoprolinase, ATP−hydrolysing
ENSMMUG00000017581	DEFB135	−17.07	3.03E−03	defensin beta 135
ENSMMUG00000045482	G0S2	−1.19	3.05E−03	G0/G1 switch 2
ENSMMUG00000004210	HTR2A	−0.76	3.07E−03	5−hydroxytryptamine receptor 2A
ENSMMUG00000003292	SLC38A10	0.57	3.07E−03	solute carrier family 38 member 10
ENSMMUG00000009048	RIMBP2	−1.93	3.10E−03	RIMS binding protein 2
ENSMMUG00000010536	DLG4	−1.03	3.13E−03	discs large MAGUK scaffold protein 4
ENSMMUG00000048898	sprn	−16.98	3.13E−03	#N/A
ENSMMUG00000002925	FSTL1	−0.35	3.16E−03	follistatin like 1
ENSMMUG00000041616	GNG11	−0.47	3.18E−03	G protein subunit gamma 11
ENSMMUG00000028736	HSD17B14	−0.83	3.19E−03	hydroxysteroid 17−beta dehydrogenase 14
ENSMMUG00000004688	NCAM1	−0.27	3.20E−03	neural cell adhesion molecule 1
ENSMMUG00000034354	5S_rRNA	16.87	3.21E−03	#N/A
ENSMMUG00000039371		−16.98	3.22E−03	#N/A
ENSMMUG00000047579	C1QTNF7	−16.93	3.22E−03	#N/A
ENSMMUG00000006111	MORF4L2	−0.43	3.23E−03	mortality factor 4 like 2
ENSMMUG00000006026	SH3D19	−0.43	3.27E−03	SH3 domain containing 19
ENSMMUG00000034102		−16.85	3.42E−03	#N/A
ENSMMUG00000039591		−16.85	3.42E−03	#N/A
ENSMMUG00000040141		−16.85	3.42E−03	#N/A
ENSMMUG00000040313		−16.85	3.42E−03	#N/A
ENSMMUG00000043077		−16.85	3.42E−03	#N/A
ENSMMUG00000045938		−16.85	3.42E−03	#N/A
ENSMMUG00000036114	7SK	−16.85	3.42E−03	#N/A
ENSMMUG00000013561	ACP6	−0.58	3.42E−03	acid phosphatase 6, lysophosphatidic
ENSMMUG00000000323	ARHGAP4	−0.87	3.42E−03	Rho GTPase activating protein 4
ENSMMUG00000010306	CLCA4	−16.85	3.42E−03	calcium−activated chloride channel regulator 4
ENSMMUG00000005964	CMAH	−0.98	3.42E−03	
ENSMMUG00000019337	FABP12	−16.92	3.42E−03	fatty acid binding protein 12
ENSMMUG00000045223	FAM110D	−16.85	3.42E−03	family with sequence similarity 110 member D
ENSMMUG00000013539	PALM3	−16.85	3.42E−03	paralemmin 3
ENSMMUG00000037196	SNORA32	−16.85	3.42E−03	#N/A
ENSMMUG00000002317	WDR91	0.72	3.42E−03	WD repeat domain 91
ENSMMUG00000044779		−16.80	3.44E−03	#N/A
ENSMMUG00000039926		−1.12	3.44E−03	#N/A
ENSMMUG00000023038	CALY	−16.85	3.44E−03	calcyon neuron specific vesicular protein
ENSMMUG00000048066	TRAPPC9	0.56	3.53E−03	trafficking protein particle complex 9
ENSMMUG00000022808	ARPC2	−0.26	3.55E−03	actin related protein 2/3 complex subunit 2

ENSMMUG00000003697	INPP4B	−0.83	3.55E−03	inositol polyphosphate−4−phosphatase type II B
ENSMMUG00000020218	AK5	−0.99	3.63E−03	adenylate kinase 5
ENSMMUG00000014982	KRT3	−16.74	3.65E−03	keratin 3
ENSMMUG00000000519	VGLL3	−0.91	3.66E−03	vestigial like family member 3
ENSMMUG00000011428	TMEM131	0.40	3.67E−03	transmembrane protein 131
ENSMMUG00000009144	NKAIN3	−16.72	3.67E−03	sodium/potassium transporting ATPase interacting 3
ENSMMUG00000003029	PRMT1	−0.44	3.71E−03	protein arginine methyltransferase 1
ENSMMUG00000013176	NSDHL	−0.53	3.74E−03	NAD(P) dependent steroid dehydrogenase−like
ENSMMUG00000030744	ARID5A	1.26	3.84E−03	AT−rich interaction domain 5A
ENSMMUG00000046550	CCDC125	−1.13	3.84E−03	coiled−coil domain containing 125
ENSMMUG00000017371	MCHR1	−0.95	3.86E−03	melanin concentrating hormone receptor 1
ENSMMUG00000021330	KYNU	−1.22	3.88E−03	kynureninase
ENSMMUG00000015733	SORBS2	−0.80	3.88E−03	sorbin and SH3 domain containing 2
ENSMMUG00000000526	ACKR4	−1.64	3.93E−03	#N/A
ENSMMUG00000031911	KRT18	−0.58	4.01E−03	keratin 18
ENSMMUG00000010164	VGF	1.50	4.04E−03	VGF nerve growth factor inducible
ENSMMUG00000048135	GRIN3B	−0.56	4.09E−03	glutamate ionotropic receptor NMDA type subunit 3B
ENSMMUG00000020664	FLRT2	−0.53	4.11E−03	#N/A
ENSMMUG00000047922		−16.58	4.15E−03	#N/A
ENSMMUG00000007052	MMP15	0.86	4.18E−03	matrix metallopeptidase 15
ENSMMUG00000000740	SPATA2	0.57	4.20E−03	spermatogenesis associated 2
ENSMMUG00000011980	ADGRG5	−16.55	4.23E−03	adhesion G protein−coupled receptor G5
ENSMMUG00000013366	NPR3	−1.48	4.23E−03	natriuretic peptide receptor 3
ENSMMUG00000021476	BCL2L13	−0.34	4.26E−03	BCL2 like 13
ENSMMUG00000010336	DOPEY2	0.43	4.26E−03	DOP1 leucine zipper like protein B
ENSMMUG00000047391	SRXN1	1.10	4.29E−03	sulfiredoxin 1
ENSMMUG00000016457	DMWD	−0.74	4.35E−03	DM1 protein kinase
ENSMMUG00000000549	AVPR1A	−16.50	4.39E−03	arginine vasopressin receptor 1A
ENSMMUG00000019820	SH3TC2	−0.63	4.66E−03	SH3 domain and tetratricopeptide repeats 2
ENSMMUG00000001016	APOBEC3H	−1.19	4.66E−03	apolipoprotein B mRNA editing enzyme catalytic subunit 3H
ENSMMUG00000047873	IFI30	−0.47	4.68E−03	#N/A
ENSMMUG00000010519	MYL1	−1.07	4.69E−03	myosin light chain 1
ENSMMUG00000012592	ME1	−0.54	4.76E−03	malic enzyme 1
ENSMMUG00000014305	APOE	−0.61	4.81E−03	apolipoprotein E
ENSMMUG00000001594	RABL3	−0.55	4.92E−03	RAB, member of RAS oncogene family like 3
ENSMMUG00000043959	MARCH2	0.59	5.04E−03	membrane associated ring−CH−type finger 2
ENSMMUG00000015546	C7orf26	0.56	5.11E−03	chromosome 3 C7orf26 homolog
ENSMMUG00000014780	C3orf20	−2.03	5.23E−03	chromosome 2 C3orf20 homolog

ENSMMUG00000035543	U6	−16.27	5.34E−03	#N/A
ENSMMUG00000004042	FCRLA	16.30	5.35E−03	Fc receptor like A
ENSMMUG00000003262	PCCB	−0.39	5.40E−03	propionyl−CoA carboxylase subunit beta
ENSMMUG00000003073	NACA	−0.47	5.73E−03	nascent polypeptide associated complex subunit alpha
ENSMMUG00000020648	ACADS	0.50	5.76E−03	acyl−CoA dehydrogenase short chain
ENSMMUG00000006768	CCDC196	−16.18	5.89E−03	coiled−coil domain containing 196
ENSMMUG00000014726	ANK1	−1.11	5.90E−03	ankyrin 1
ENSMMUG00000022037	LDHD	−1.03	5.96E−03	lactate dehydrogenase D
ENSMMUG00000000168	MGLL	−0.65	5.99E−03	monoglyceride lipase
ENSMMUG00000006243	TDRD9	−16.12	6.01E−03	tudor domain containing 9
ENSMMUG00000023318	TRRAP	0.29	6.01E−03	transformation/transcription domain associated protein
ENSMMUG00000008547	FAM198B	−0.97	6.04E−03	golgi associated kinase 1B
ENSMMUG00000004600	RCAN1	0.97	6.08E−03	regulator of calcineurin 1
ENSMMUG00000009156	LIMS1	−0.51	6.18E−03	LIM zinc finger domain containing 1
ENSMMUG00000046558		−16.12	6.18E−03	#N/A
ENSMMUG00000023986	Y_RNA	16.12	6.19E−03	Y RNA
ENSMMUG00000022992	C8A	16.07	6.19E−03	complement C8 alpha chain
ENSMMUG00000000022	ZP1	16.06	6.19E−03	zona pellucida glycoprotein 1
ENSMMUG00000011574	CAVIN2	−1.74	6.23E−03	caveolae associated protein 2
ENSMMUG00000019067	IFNB1	−16.05	6.26E−03	interferon beta 1
ENSMMUG00000013445	LOC694492	−16.10	6.28E−03	neuropeptide Y receptor type 4
ENSMMUG00000014182	SERPINB5	−2.00	6.44E−03	serpin family B member 5
ENSMMUG00000022087	PRAG1	0.94	6.57E−03	PEAK1 related, kinase−activating pseudokinase 1
ENSMMUG00000012324		0.67	6.82E−03	#N/A
ENSMMUG00000012109	TSPAN33	1.69	6.82E−03	tetraspanin 33
ENSMMUG00000015293	CLEC2D	−4.34	6.84E−03	C−type lectin domain family 2 member D
ENSMMUG00000000754	DMXL2	1.52	6.85E−03	Dmx like 2
ENSMMUG00000021247	ALS2CL	−0.69	6.95E−03	ALS2 C−terminal like
ENSMMUG00000017819	PHTF1	0.26	6.99E−03	putative homeodomain transcription factor 1
ENSMMUG00000004918	ZFAT	−0.87	7.09E−03	zinc finger and AT−hook domain containing
ENSMMUG00000047162	FGF10	−1.82	7.13E−03	fibroblast growth factor 10
ENSMMUG00000005093	KLHL32	−0.93	7.13E−03	kelch like family member 32
ENSMMUG00000012036	RBM47	−0.37	7.32E−03	RNA binding motif protein 47
ENSMMUG00000020322	ALDOC	−0.98	7.37E−03	aldolase, fructose−bisphosphate C
ENSMMUG00000003448	RPL26	−0.30	7.37E−03	ribosomal protein L26
ENSMMUG00000038719	SOD2	−0.71	7.52E−03	#N/A
ENSMMUG00000003718	TLE1	0.51	7.52E−03	TLE family member 1, transcriptional corepressor
ENSMMUG00000012588	HDAC3	0.51	7.64E−03	histone deacetylase 3

ENSMMUG00000012972	ULK3	0.49	7.70E−03	unc−51 like kinase 3
ENSMMUG00000004907	FAM46C	2.43	7.71E−03	terminal nucleotidyltransferase 5C
ENSMMUG00000009937	PIPOX	−0.92	7.79E−03	pipecolic acid and sarcosine oxidase
ENSMMUG00000020066	MCF2	−15.82	7.94E−03	MCF.2 cell line derived transforming sequence
ENSMMUG00000045847	PAGE2B	−15.76	7.97E−03	PAGE family member 2B
ENSMMUG00000000963	SDC2	−0.44	7.97E−03	syndecan 2
ENSMMUG00000021374	EWSR1	−0.25	8.09E−03	EWS RNA binding protein 1
ENSMMUG00000012138	RORC	−0.73	8.15E−03	RAR related orphan receptor C
ENSMMUG00000010442	NRDE2	−0.39	8.15E−03	NRDE−2, necessary for RNA interference, domain containing
ENSMMUG00000007678	ANKRD13A	0.40	8.19E−03	ankyrin repeat domain 13A
ENSMMUG00000014230	FMO4	−0.53	8.22E−03	flavin containing dimethylaniline monoxygenase 4
ENSMMUG00000002781	PXDNL	−0.49	8.22E−03	peroxidasin like
ENSMMUG00000021436	LTBP1	1.20	8.25E−03	latent transforming growth factor beta binding protein 1
ENSMMUG00000011115	TYR	−0.81	8.29E−03	tyrosinase
ENSMMUG00000043239	MIR4736	15.68	8.47E−03	microRNA 4736
ENSMMUG00000018360	RCN1	−0.38	8.47E−03	reticulocalbin 1
ENSMMUG00000007246	MTMR11	−0.74	8.54E−03	myotubularin related protein 11
ENSMMUG00000045520	RASGRP3	−0.78	8.54E−03	RAS guanyl releasing protein 3
ENSMMUG00000014123	PTGES3L−AARSD1	−0.52	8.55E−03	alanyl−tRNA editing protein Aarsd1
ENSMMUG00000002712	SCN2A	15.66	8.56E−03	sodium channel protein type 2 subunit alpha
ENSMMUG00000037338	SNORA35	15.66	8.56E−03	#N/A
ENSMMUG00000004415	TNIP3	−1.35	8.61E−03	TNFAIP3 interacting protein 3
ENSMMUG00000011835	TRPC6	−0.78	8.62E−03	transient receptor potential cation channel subfamily C member 6
ENSMMUG00000032643	mml−mir−675	−1.20	8.83E−03	mml−mir−675
ENSMMUG00000011714	ZFP91	−0.36	8.93E−03	ZFP91 zinc finger protein
ENSMMUG00000029510	SERAC1	−0.47	8.96E−03	serine active site containing 1
ENSMMUG00000018411	INSL4	−1.26	9.04E−03	#N/A
ENSMMUG00000007376	ADCK5	0.39	9.08E−03	aarF domain containing kinase 5
ENSMMUG00000000194	SLC27A1	0.50	9.08E−03	solute carrier family 27 member 1
ENSMMUG00000022573	DIRC2	0.44	9.12E−03	solute carrier family 49 member 4
ENSMMUG00000005782	EPHX1	0.77	9.12E−03	epoxide hydrolase 1
ENSMMUG00000005601	OPTN	−0.35	9.12E−03	optineurin
ENSMMUG00000007959	PCYOX1	0.36	9.12E−03	prenylcysteine oxidase 1
ENSMMUG00000047311		−0.52	9.16E−03	#N/A
ENSMMUG00000049102	C8orf88	−0.89	9.16E−03	chromosome 8 C8orf88 homolog
ENSMMUG00000016321	TPM2	−0.77	9.20E−03	tropomyosin 2
ENSMMUG00000040221		0.41	9.28E−03	#N/A
ENSMMUG00000014720	NCKIPSD	0.45	9.28E−03	NCK interacting protein with SH3 domain

ENSMMUG00000010225	RAB11B	0.73	9.28E−03	RAB11B, member RAS oncogene family
ENSMMUG00000005527	PPM1F	0.51	9.33E−03	protein phosphatase, Mg2+/Mn2+ dependent 1F
ENSMMUG00000001537	APOL2	−0.69	9.37E−03	apolipoprotein L2
ENSMMUG00000043241		0.96	9.54E−03	#N/A
ENSMMUG00000047793	ENDOD1	0.46	9.54E−03	endonuclease domain containing 1
ENSMMUG00000005214	MYL6B	−0.39	9.63E−03	myosin light chain 6B
ENSMMUG00000019611	ARRDC1	0.45	9.67E−03	arrestin domain containing 1
ENSMMUG00000022865	JPH2	−0.72	9.73E−03	junctophilin 2
ENSMMUG00000022246	PDLIM5	−0.43	9.73E−03	PDZ and LIM domain 5
ENSMMUG00000002847	REXO1	0.54	9.73E−03	RNA exonuclease 1 homolog
ENSMMUG00000000419	ARHGAP26	−0.58	9.79E−03	Rho GTPase activating protein 26
ENSMMUG00000014143	SARAF	0.29	9.89E−03	store−operated calcium entry associated regulatory factor
ENSMMUG00000009188	CAP1	−0.33	9.97E−03	cyclase associated actin cytoskeleton regulatory protein 1
ENSMMUG00000019742	ZNF512	−0.38	9.99E−03	zinc finger protein 512
ENSMMUG00000013331	GDE1	0.41	1.00E−02	glycerophosphodiester phosphodiesterase 1
ENSMMUG00000005633	MMADHC	−0.37	1.01E−02	metabolism of cobalamin associated D
ENSMMUG00000021183	ISYNA1	−0.49	1.01E−02	inositol−3−phosphate synthase 1
ENSMMUG00000014833	IDH2	−0.61	1.02E−02	isocitrate dehydrogenase (NADP(+)) 2
ENSMMUG00000018087	TSPOAP1	−0.49	1.02E−02	TSPO associated protein 1
ENSMMUG00000020582	RPL38	−0.34	1.03E−02	ribosomal protein L38
ENSMMUG00000011870	RPS7	−0.28	1.03E−02	ribosomal protein S7
ENSMMUG00000017932	ACSS2	−0.55	1.03E−02	acyl−CoA synthetase short chain family member 2
ENSMMUG00000015386	SLITRK4	−15.39	1.04E−02	#N/A
ENSMMUG00000012349	TST	0.70	1.05E−02	thiosulfate sulfurtransferase
ENSMMUG00000016308	UNC13B	0.26	1.05E−02	unc−13 homolog B
ENSMMUG00000018001	NDP	−1.52	1.06E−02	norrin cystine knot growth factor NDP
ENSMMUG00000035188	5S_rRNA	−15.34	1.08E−02	#N/A
ENSMMUG00000021534	SLC16A10	15.30	1.08E−02	solute carrier family 16 member 10
ENSMMUG00000038976		−1.39	1.09E−02	#N/A
ENSMMUG00000000484	C2CD4C	−2.20	1.09E−02	#N/A
ENSMMUG00000039441	MAB21L2	−0.74	1.09E−02	mab−21 like 2
ENSMMUG00000022882	HES2	−0.64	1.10E−02	hes family bHLH transcription factor 2
ENSMMUG00000019390	TMEM184B	0.40	1.10E−02	#N/A
ENSMMUG00000003291	TEPSIN	0.53	1.11E−02	TEPSIN adaptor related protein complex 4 accessory protein
ENSMMUG00000016264	ALOX12	0.63	1.11E−02	arachidonate 12−lipoxygenase, 12S type
ENSMMUG00000012449	SYTL2	−0.58	1.11E−02	synaptotagmin like 2
ENSMMUG00000014703	HACE1	−0.72	1.12E−02	HECT domain and ankyrin repeat containing E3 ubiquitin protein ligase 1
ENSMMUG00000002854	SLITRK5	−0.96	1.12E−02	SLIT and NTRK like family member 5

ENSMMUG00000010556	SRCAP	0.45	1.12E−02	Snf2 related CREBBP activator protein
ENSMMUG00000002063	ERAP1	−0.37	1.13E−02	endoplasmic reticulum aminopeptidase 1
ENSMMUG00000010405	FAM171B	1.09	1.13E−02	family with sequence similarity 171 member B
ENSMMUG00000001226	HMGCS1	−0.87	1.13E−02	3−hydroxy−3−methylglutaryl−CoA synthase 1
ENSMMUG00000011038	FZD2	−0.62	1.14E−02	frizzled class receptor 2
ENSMMUG00000011513	VCAN	−1.18	1.14E−02	versican
ENSMMUG00000018621	CDK7	−0.56	1.16E−02	cyclin dependent kinase 7
ENSMMUG00000018198	CCDC6	−0.32	1.17E−02	coiled−coil domain containing 6
ENSMMUG00000037980	CDKN1C	−2.15	1.18E−02	cyclin dependent kinase inhibitor 1C
ENSMMUG00000022644	ATP6V0A1	0.37	1.18E−02	ATPase H+ transporting V0 subunit a1
ENSMMUG00000008691	VDAC2	−0.23	1.18E−02	voltage dependent anion channel 2
ENSMMUG00000015260	PRSS12	−0.53	1.19E−02	serine protease 12
ENSMMUG00000002811	SNTG1	−2.48	1.19E−02	syntrophin gamma 1
ENSMMUG00000029821		0.38	1.19E−02	#N/A
ENSMMUG00000023702	SEMA6B	−0.49	1.19E−02	semaphorin 6B
ENSMMUG00000031367		−0.47	1.20E−02	
ENSMMUG00000035344	5S_rRNA	15.11	1.20E−02	#N/A
ENSMMUG00000012364	EPHA4	15.12	1.20E−02	EPH receptor A4
ENSMMUG00000001062	EIF4B	−0.34	1.21E−02	eukaryotic translation initiation factor 4B
ENSMMUG00000000188	NXF1	0.45	1.21E−02	nuclear RNA export factor 1
ENSMMUG00000019358	AHCYL2	0.39	1.22E−02	adenosylhomocysteinase like 2
ENSMMUG00000010499	C3orf18	−1.73	1.23E−02	chromosome 2 C3orf18 homolog
ENSMMUG00000016180	MRPS30	−0.45	1.26E−02	mitochondrial ribosomal protein S30
ENSMMUG00000006066	PCTP	0.73	1.26E−02	phosphatidylcholine transfer protein
ENSMMUG00000020558	STK38	−0.39	1.27E−02	serine/threonine kinase 38
ENSMMUG00000001134	CDKN2A	−0.62	1.27E−02	cyclin−dependent kinase inhibitor 2A
ENSMMUG00000018571	CREB3L1	−0.68	1.29E−02	cAMP responsive element binding protein 3 like 1
ENSMMUG00000022909	MGAT4A	2.32	1.29E−02	alpha−1,3−mannosyl−glycoprotein 4−beta−N−acetylglucosaminyltransferase A
ENSMMUG00000044474	TNFRSF12A	0.45	1.29E−02	TNF receptor superfamily member 12A
ENSMMUG00000015059	RPL24	−0.28	1.29E−02	ribosomal protein L24
ENSMMUG00000003755	ITGA5	−0.64	1.29E−02	integrin subunit alpha 5
ENSMMUG00000021536	OLFML3	−1.65	1.31E−02	#N/A
ENSMMUG00000008203	RHOJ	−1.21	1.32E−02	ras homolog family member J
ENSMMUG00000022964	DDB2	−0.66	1.32E−02	
ENSMMUG00000045396	SELENBP1	−0.68	1.32E−02	selenium binding protein 1
ENSMMUG00000016754	TLR1	−1.82	1.35E−02	toll like receptor 1
ENSMMUG00000039955	MSMO1	−0.61	1.35E−02	#N/A
ENSMMUG00000003563		14.94	1.36E−02	#N/A

ENSMMUG00000004300	NCAM2	14.94	1.36E−02	neural cell adhesion molecule 2
ENSMMUG00000003041	MYO5B	−0.58	1.37E−02	myosin VB
ENSMMUG00000014863	FAM174A	0.47	1.37E−02	#N/A
ENSMMUG00000012235	CD274	−0.82	1.39E−02	CD274 molecule
ENSMMUG00000015396	WDR13	−0.49	1.40E−02	WD repeat domain 13
ENSMMUG00000000951	ITGB2	−0.36	1.41E−02	integrin subunit beta 2
ENSMMUG00000015317	HDDC2	−0.36	1.42E−02	HD domain containing 2
ENSMMUG00000020394	CCDC102A	−0.48	1.43E−02	coiled−coil domain containing 102A
ENSMMUG00000019684	NQO1	0.46	1.44E−02	NAD(P)H quinone dehydrogenase 1
ENSMMUG00000018371	RALGAPA2	0.36	1.44E−02	Ral GTPase activating protein catalytic alpha subunit 2
ENSMMUG00000015142	IPO13	0.37	1.44E−02	importin 13
ENSMMUG00000004657	FURIN	0.47	1.45E−02	furin, paired basic amino acid cleaving enzyme
ENSMMUG00000003100	GMNC	−1.63	1.47E−02	#N/A
ENSMMUG00000015123	CDAN1	0.47	1.48E−02	codanin 1
ENSMMUG00000016975	SLC38A5	−1.50	1.49E−02	solute carrier family 38 member 5
ENSMMUG00000009384	IGFBP4	−0.59	1.50E−02	insulin like growth factor binding protein 4
ENSMMUG00000007627	SUSD5	1.13	1.50E−02	sushi domain containing 5
ENSMMUG00000040683	ANKRD63	14.78	1.51E−02	ankyrin repeat domain 63
ENSMMUG00000007576	EYA1	−0.80	1.51E−02	EYA transcriptional coactivator and phosphatase 1
ENSMMUG00000015256	FOXJ2	0.34	1.51E−02	forkhead box J2
ENSMMUG00000013101	TAF3	0.57	1.52E−02	TATA−box binding protein associated factor 3
ENSMMUG00000010864	STAMBP	−0.35	1.52E−02	STAM binding protein
ENSMMUG00000019489	MAOA	−0.47	1.53E−02	monoamine oxidase A
ENSMMUG00000003013	NIPA1	0.55	1.55E−02	NIPA magnesium transporter 1
ENSMMUG00000003708	PDE7B	−1.16	1.57E−02	phosphodiesterase 7B
ENSMMUG00000001940	NME1−NME2	−0.38	1.58E−02	NME/NM23 nucleoside diphosphate kinase 2
ENSMMUG00000012293	CDH10	−1.50	1.60E−02	cadherin 10
ENSMMUG00000017841	DCAF8	0.28	1.61E−02	DDB1 and CUL4 associated factor 8
ENSMMUG00000018109	MINDY2	−0.62	1.61E−02	MINDY lysine 48 deubiquitinase 2
ENSMMUG00000044175	FOXO6	−1.00	1.62E−02	forkhead box O6
ENSMMUG00000003591	PLEKHA2	−0.40	1.62E−02	pleckstrin homology domain containing A2
ENSMMUG00000020365	ARHGAP18	−0.72	1.63E−02	Rho GTPase activating protein 18
ENSMMUG00000008702	SYNPO2	−0.74	1.63E−02	synaptopodin 2
ENSMMUG00000011264	MFSD2A	−1.36	1.63E−02	major facilitator superfamily domain containing 2A
ENSMMUG00000013997	PLIN2	0.70	1.63E−02	perilipin−2
ENSMMUG00000004902	SLC25A44	0.39	1.63E−02	bone gamma−carboxyglutamate protein
ENSMMUG00000043224		−0.58	1.64E−02	#N/A
ENSMMUG00000019118	ST13	−0.21	1.64E−02	ST13 Hsp70 interacting protein

ENSMMUG00000020736	HACD1	−0.55	1.65E−02	3−hydroxyacyl−CoA dehydratase 1
ENSMMUG00000039715	H19_1	−1.18	1.70E−02	#N/A
ENSMMUG00000009343	ICAM4	−0.79	1.70E−02	intercellular adhesion molecule 4 (Landsteiner−Wiener blood group)
ENSMMUG00000022732	SRPK2	−0.38	1.70E−02	SRSF protein kinase 2
ENSMMUG00000003902	THBS3	−0.34	1.73E−02	thrombospondin 3
ENSMMUG00000014111	NR5A1	14.60	1.74E−02	nuclear receptor subfamily 5 group A member 1
ENSMMUG00000002359	MGST1	−0.49	1.76E−02	#N/A
ENSMMUG00000038237		−1.63	1.77E−02	#N/A
ENSMMUG00000044591		−0.70	1.78E−02	#N/A
ENSMMUG00000018404	GAL3ST4	−1.13	1.78E−02	galactose−3−O−sulfotransferase 4
ENSMMUG00000014256	TMSB4X	−0.40	1.78E−02	thymosin beta 4 X−linked
ENSMMUG00000016931	KCNK2	−0.70	1.80E−02	potassium two pore domain channel subfamily K member 2
ENSMMUG00000019826	MAZ	−0.44	1.81E−02	MYC associated zinc finger protein
ENSMMUG00000018551	ANGEL1	0.29	1.82E−02	angel homolog 1
ENSMMUG00000016187	SYBU	−1.21	1.82E−02	syntabulin
ENSMMUG00000004885	QSOX2	0.45	1.82E−02	quiescin sulfhydryl oxidase 2
ENSMMUG00000004638	ASPN	−2.61	1.83E−02	asporin
ENSMMUG00000001938	CTC1	0.60	1.83E−02	CST telomere replication complex component 1
ENSMMUG00000023375	SREBF1	−0.79	1.83E−02	sterol regulatory element binding transcription factor 1
ENSMMUG00000005035		14.49	1.87E−02	#N/A
ENSMMUG00000014523	ITIH4	14.49	1.87E−02	inter−alpha−trypsin inhibitor heavy chain 4
ENSMMUG00000027367	Y_RNA	14.49	1.87E−02	Y RNA
ENSMMUG00000041505		14.47	1.90E−02	#N/A
ENSMMUG00000006943	ZNF706	−0.35	1.94E−02	zinc finger protein 706
ENSMMUG00000011951	KRI1	0.60	1.96E−02	KRI1 homolog
ENSMMUG00000015849	RNF175	−1.43	1.98E−02	ring finger protein 175
ENSMMUG00000019836	MEF2C	−1.36	2.00E−02	myocyte enhancer factor 2C
ENSMMUG00000000458	RBPMS	−0.38	2.00E−02	RNA binding protein, mRNA processing factor
ENSMMUG00000002739	PDGFB	−0.88	2.05E−02	platelet derived growth factor subunit B
ENSMMUG00000016139	FLT3	−14.42	2.05E−02	fms related tyrosine kinase 3
ENSMMUG00000011126	DBI	−0.39	2.06E−02	diazepam binding inhibitor, acyl−CoA binding protein
ENSMMUG00000007039	COTL1	−0.48	2.07E−02	#N/A
ENSMMUG00000016067	RSU1	−0.35	2.07E−02	Ras suppressor protein 1
ENSMMUG00000015832	NSMCE1	−0.53	2.10E−02	NSE1 homolog, SMC5−SMC6 complex component
ENSMMUG00000016350	RPS27L	−0.38	2.10E−02	ribosomal protein S27 like
ENSMMUG00000038082		0.72	2.11E−02	#N/A
ENSMMUG00000004869	NPTXR	1.02	2.12E−02	neuronal pentraxin receptor
ENSMMUG00000012924	ANO3	−3.25	2.15E−02	anoctamin 3

ENSMMUG00000012001	TCF4	−0.59	2.15E−02	transcription factor 4
ENSMMUG00000019136	TXLNB	−1.27	2.15E−02	taxilin beta
ENSMMUG00000013166	ZNF687	0.41	2.17E−02	zinc finger protein 687
ENSMMUG00000014257	FAM83H	−0.58	2.18E−02	family with sequence similarity 83 member H
ENSMMUG00000038190	CYP51A1	−0.47	2.18E−02	Cytochrome P450 Family 51 Subfamily A Member 1
ENSMMUG00000012811	FASN	−0.62	2.18E−02	fatty acid synthase
ENSMMUG00000048816	HAND2	−1.85	2.18E−02	heart and neural crest derivatives expressed 2
ENSMMUG00000006617	LBH	0.84	2.18E−02	LBH regulator of WNT signaling pathway
ENSMMUG00000022283	ATRN	0.27	2.24E−02	attractin
ENSMMUG00000012895	PYCR1	−0.90	2.24E−02	pyrroline−5−carboxylate reductase 1
ENSMMUG00000002917	SIGIRR	−1.57	2.25E−02	single Ig IL−1−related receptor
ENSMMUG00000036913	U7	14.23	2.25E−02	U7 small nuclear RNA
ENSMMUG00000048077	ATXN1L	0.26	2.25E−02	ataxin 1 like
ENSMMUG00000013324	GPRC5C	−0.62	2.28E−02	G protein−coupled receptor class C group 5 member C
ENSMMUG00000048418	PPDPF	0.70	2.29E−02	pancreatic progenitor cell differentiation and proliferation factor
ENSMMUG00000018232	SESN1	−0.40	2.29E−02	sestrin 1
ENSMMUG00000007557		0.61	2.29E−02	#N/A
ENSMMUG00000010246	TTI2	0.31	2.30E−02	#N/A
ENSMMUG00000008724	PPAPDC1A	−0.77	2.31E−02	phospholipid phosphatase 4
ENSMMUG00000048545	SUMO3	−0.48	2.31E−02	small ubiquitin like modifier 3
ENSMMUG00000013193	MMP16	−0.53	2.31E−02	matrix metallopeptidase 16
ENSMMUG00000000250	COX7A1	−0.49	2.32E−02	cytochrome c oxidase subunit 7A1
ENSMMUG00000043393		−14.17	2.36E−02	#N/A
ENSMMUG00000003998	PPP1R12B	−0.59	2.39E−02	protein phosphatase 1 regulatory subunit 12B
ENSMMUG00000049145	CKB	−1.68	2.40E−02	creatine kinase B
ENSMMUG00000001593	HGD	−2.81	2.42E−02	homogentisate 1,2−dioxygenase
ENSMMUG00000014717	HGH1	0.52	2.42E−02	HGH1 homolog
ENSMMUG00000002517	PTPDC1	−3.64	2.46E−02	protein tyrosine phosphatase domain containing 1
ENSMMUG00000002589	SULT1C3	14.09	2.46E−02	sulfotransferase 1C3−like
ENSMMUG00000031865	TMEM8B	1.03	2.46E−02	transmembrane protein 8B
ENSMMUG00000039499		14.11	2.46E−02	#N/A
ENSMMUG00000045408	H19_2	−1.51	2.49E−02	#N/A
ENSMMUG00000003502	TCEAL4	−0.38	2.51E−02	transcription elongation factor A like 4
ENSMMUG00000008265	GULP1	−1.29	2.52E−02	GULP PTB domain containing engulfment adaptor 1
ENSMMUG00000001029	ANO10	0.26	2.53E−02	anoctamin 10
ENSMMUG00000000149	KCTD17	0.42	2.54E−02	potassium channel tetramerization domain containing 17
ENSMMUG00000013405	ADAMTS5	−0.96	2.54E−02	ADAM metallopeptidase with thrombospondin type 1 motif 5
ENSMMUG00000012132	PDE4D	−0.84	2.54E−02	phosphodiesterase 4D

ENSMMUG00000020625		0.82	2.54E−02	#N/A
ENSMMUG00000002945	FILIP1L	−0.89	2.56E−02	filamin A interacting protein 1 like
ENSMMUG00000017134	SEPT5	−0.63	2.56E−02	septin 5
ENSMMUG00000023669	MAD2L2	0.45	2.57E−02	mitotic arrest deficient 2 like 2
ENSMMUG00000021135	SLC34A2	−2.92	2.58E−02	solute carrier family 34 member 2
ENSMMUG00000002183	OSBPL6	0.55	2.59E−02	oxysterol binding protein like 6
ENSMMUG00000016845	ZEB2	−0.61	2.59E−02	zinc finger E−box binding homeobox 2
ENSMMUG00000046927	IGKC	−1.07	2.60E−02	#N/A
ENSMMUG00000015710	ZMYND8	−0.45	2.61E−02	zinc finger MYND−type containing 8
ENSMMUG00000042653	ZFP36L2	0.50	2.61E−02	#N/A
ENSMMUG00000038179	NRXN1	−14.03	2.63E−02	neurexin 1
ENSMMUG00000041958	BHLHA15	−3.79	2.65E−02	#N/A
ENSMMUG00000006758	CYP27A1	−0.93	2.69E−02	cytochrome P450, family 27, subfamily A, polypeptide 1
ENSMMUG00000018627	TXK	13.94	2.69E−02	#N/A
ENSMMUG00000013407	ADAMTS1	−0.96	2.71E−02	ADAM metallopeptidase with thrombospondin type 1 motif 1
ENSMMUG00000008120	CRYBG1	−0.38	2.73E−02	crystallin beta−gamma domain containing 1
ENSMMUG00000001731	GFPT2	1.27	2.73E−02	glutamine−fructose−6−phosphate transaminase 2
ENSMMUG00000018123	PPP1R13B	0.43	2.73E−02	protein phosphatase 1 regulatory subunit 13B
ENSMMUG00000001313	FARP2	0.38	2.75E−02	FERM, ARH/RhoGEF and pleckstrin domain protein 2
ENSMMUG00000023218	ONECUT2	−13.96	2.76E−02	one cut homeobox 2
ENSMMUG00000007838	PRCP	0.39	2.76E−02	prolylcarboxypeptidase
ENSMMUG00000040809	VAMP1	−0.51	2.76E−02	#N/A
ENSMMUG00000009690	ARNTL	−1.03	2.78E−02	aryl hydrocarbon receptor nuclear translocator like
ENSMMUG00000014633	ETV4	−0.91	2.78E−02	ETS variant 4
ENSMMUG00000021629	LENG8	0.42	2.78E−02	leukocyte receptor cluster member 8
ENSMMUG00000012297	PARP4	0.23	2.83E−02	protein mono−ADP−ribosyltransferase PARP4
ENSMMUG00000023802	SAMD4B	0.40	2.83E−02	sterile alpha motif domain containing 4B
ENSMMUG00000021796	CHRNA1	−1.17	2.84E−02	cholinergic receptor nicotinic alpha 1 subunit
ENSMMUG00000005580		−2.94	2.86E−02	#N/A
ENSMMUG00000005858	SPATA5L1	0.57	2.86E−02	spermatogenesis associated 5 like 1
ENSMMUG00000003143	THBS4	−13.90	2.86E−02	thrombospondin 4
ENSMMUG00000042820		−13.89	2.87E−02	#N/A
ENSMMUG00000002861	TMEM45A	−0.67	2.87E−02	transmembrane protein 45A
ENSMMUG00000021833	SLC25A25	−0.40	2.88E−02	solute carrier family 25 member 25
ENSMMUG00000017259	DNAJC6	−0.41	2.90E−02	DnaJ heat shock protein family (Hsp40) member C6
ENSMMUG00000015084	LAMC1	−0.32	2.90E−02	laminin subunit gamma 1
ENSMMUG00000023452	CRYBB1	13.85	2.90E−02	crystallin beta B1
ENSMMUG00000015570	KIAA0556	0.40	2.95E−02	KIAA0556

ENSMMUG00000017701	MYRIP	−0.83	2.95E−02	myosin VIIA and Rab interacting protein
ENSMMUG00000015133	CD34	−13.84	2.96E−02	CD34 molecule
ENSMMUG00000010661	SEC61B	−0.44	2.96E−02	SEC61 translocon beta subunit
ENSMMUG00000019266	CHDH	−0.47	2.97E−02	choline dehydrogenase
ENSMMUG00000004236	NAPEPLD	−0.50	2.97E−02	N−acyl phosphatidylethanolamine phospholipase D
ENSMMUG00000020274	TAOK2	0.31	2.99E−02	TAO kinase 2
ENSMMUG00000019368	ALDH5A1	0.55	3.00E−02	aldehyde dehydrogenase 5 family member A1
ENSMMUG00000011841	THUMPD2	−0.52	3.00E−02	THUMP domain containing 2
ENSMMUG00000014753	FAM220A	0.29	3.00E−02	small integral membrane protein 10−like protein 2A
ENSMMUG00000014742	PTH1R	−1.77	3.00E−02	parathyroid hormone 1 receptor
ENSMMUG00000006391	TYSND1	0.48	3.00E−02	trypsin domain containing 1
ENSMMUG00000014326	MVD	−0.66	3.06E−02	mevalonate diphosphate decarboxylase
ENSMMUG00000008105	GPR75	0.45	3.07E−02	G protein−coupled receptor 75
ENSMMUG00000000338	TRIM22	−0.97	3.08E−02	tripartite motif containing 22
ENSMMUG00000007565	CBLB	−0.36	3.09E−02	Cbl proto−oncogene B
ENSMMUG00000006687	SMARCD3	−0.51	3.11E−02	SWI/SNF related, matrix associated, actin dependent regulator of chromatin, subfamily d, member 3
ENSMMUG00000001923	LMOD1	−0.90	3.15E−02	leiomodin 1
ENSMMUG00000003846	EHD4	−0.34	3.18E−02	EH domain containing 4
ENSMMUG00000031296	CASP8	−0.36	3.20E−02	caspase 8
ENSMMUG00000006867	MYPN	−1.58	3.20E−02	myopalladin
ENSMMUG00000004303	KDELR3	−0.59	3.21E−02	KDEL endoplasmic reticulum protein retention receptor 3
ENSMMUG00000017483	EPS8L2	−0.73	3.22E−02	EPS8 like 2
ENSMMUG00000020476	P2RX6	−1.42	3.23E−02	purinergic receptor P2X 6
ENSMMUG00000000118	PLAGL2	0.45	3.23E−02	PLAG1 like zinc finger 2
ENSMMUG00000012473	PSME2	−0.39	3.23E−02	proteasome activator subunit 2
ENSMMUG00000000789	COX18	−0.36	3.23E−02	cytochrome c oxidase assembly factor COX18
ENSMMUG00000014299	C2orf80	13.67	3.23E−02	chromosome 12 C2orf80 homolog
ENSMMUG00000001819		−0.87	3.25E−02	#N/A
ENSMMUG00000015777	HNRNPDL	−0.53	3.28E−02	heterogeneous nuclear ribonucleoprotein D like
ENSMMUG00000004596	GRK5	1.33	3.30E−02	G protein−coupled receptor kinase 5
ENSMMUG00000020113	CMBL	−0.48	3.30E−02	carboxymethylenebutenolidase homolog
ENSMMUG00000042891	QPRT	−0.89	3.30E−02	quinolinate phosphoribosyltransferase
ENSMMUG00000014010	RBFOX2	−0.66	3.30E−02	RNA binding fox−1 homolog 2
ENSMMUG00000002374		0.37	3.32E−02	#N/A
ENSMMUG00000006231	FAM107A	−2.01	3.33E−02	#N/A
ENSMMUG00000005052	FBXL18	0.57	3.33E−02	#N/A
ENSMMUG00000038836	SMIM1	−0.63	3.33E−02	small integral membrane protein 1 (Vel blood group)
ENSMMUG00000002672	TMEM38B	0.70	3.35E−02	transmembrane protein 38B

ENSMMUG00000007433	RPL6	−0.20	3.36E−02	ribosomal protein L6
ENSMMUG00000011023	PANX2	−13.63	3.36E−02	pannexin 2
ENSMMUG00000002675	CAPG	−0.41	3.38E−02	capping actin protein, gelsolin like
ENSMMUG00000009852	CASP1	−0.86	3.38E−02	caspase−1
ENSMMUG00000016270	ANAPC15	−0.49	3.39E−02	#N/A
ENSMMUG00000009199	PYGB	0.37	3.39E−02	glycogen phosphorylase B
ENSMMUG00000012102	SLC22A31	−13.61	3.40E−02	solute carrier family 22 member 31
ENSMMUG00000005790	TRIM25	0.53	3.43E−02	tripartite motif containing 25
ENSMMUG00000011907	ZNF488	−0.75	3.46E−02	
ENSMMUG00000039030	GSR	0.32	3.48E−02	glutathione−disulfide reductase
ENSMMUG00000007284	SUN2	−0.46	3.50E−02	Sad1 and UNC84 domain containing 2
ENSMMUG00000003494	RBM6	0.28	3.50E−02	RNA binding motif protein 6
ENSMMUG00000006906	LPAR1	−0.97	3.51E−02	lysophosphatidic acid receptor 1
ENSMMUG00000017336	PDHA1	−0.32	3.52E−02	pyruvate dehydrogenase E1 alpha 1 subunit
ENSMMUG00000007752	PHLDA2	−0.80	3.53E−02	pleckstrin homology like domain family A member 2
ENSMMUG00000006147	AGPAT4	−0.67	3.53E−02	1−acylglycerol−3−phosphate O−acyltransferase 4
ENSMMUG00000003511	CLIC5	−0.93	3.53E−02	chloride intracellular channel 5
ENSMMUG00000008101	SPATS2L	−0.28	3.53E−02	spermatogenesis associated serine rich 2 like
ENSMMUG00000004255	YLPM1	0.31	3.53E−02	YLP motif containing 1
ENSMMUG00000005430	NR2F2	−0.44	3.55E−02	nuclear receptor subfamily 2 group F member 2
ENSMMUG00000017360	NT5DC2	−0.47	3.56E−02	5'−nucleotidase domain containing 2
ENSMMUG00000020074	PKDCC	0.58	3.59E−02	protein kinase domain containing, cytoplasmic
ENSMMUG00000022253	CCNDBP1	0.44	3.59E−02	cyclin D1 binding protein 1
ENSMMUG00000000662	GBP2	−0.63	3.60E−02	guanylate−binding protein 1
ENSMMUG00000009332	HSD17B7	−0.28	3.60E−02	3−keto−steroid reductase
ENSMMUG00000021730	SEMA3D	−1.00	3.60E−02	semaphorin 3D
ENSMMUG00000019330	RBMS2	−0.45	3.60E−02	RNA binding motif single stranded interacting protein 2
ENSMMUG00000015267	CSK	0.42	3.62E−02	C−terminal Src kinase
ENSMMUG00000009366	DOCK2	−0.33	3.62E−02	dedicator of cytokinesis 2
ENSMMUG00000006069	FNDC11	−0.81	3.62E−02	fibronectin type III domain containing 11
ENSMMUG00000013257		−0.49	3.62E−02	
ENSMMUG00000048425		0.24	3.64E−02	#N/A
ENSMMUG00000047461		−0.28	3.66E−02	#N/A
ENSMMUG00000023018	RP2	−0.75	3.70E−02	RP2 activator of ARL3 GTPase
ENSMMUG00000020935	SLC12A6	0.32	3.72E−02	solute carrier family 12 member 6
ENSMMUG00000018671	CLDN16	−1.46	3.74E−02	claudin 16
ENSMMUG00000010280	SKI	0.59	3.74E−02	SKI proto−oncogene
ENSMMUG00000018289	PRRT3	0.85	3.77E−02	proline rich transmembrane protein 3

ENSMMUG00000017040	PRDM2	0.35	3.77E−02	PR/SET domain 2
ENSMMUG00000023286	UPF1	0.30	3.81E−02	UPF1 RNA helicase and ATPase
ENSMMUG00000015774	NPM3	−0.40	3.83E−02	nucleophosmin/nucleoplasmin 3
ENSMMUG00000006257	ECM1	−0.34	3.83E−02	extracellular matrix protein 1
ENSMMUG00000003046	GAMT	0.54	3.84E−02	guanidinoacetate N−methyltransferase
ENSMMUG00000002975	DUSP14	0.49	3.84E−02	dual specificity phosphatase 14
ENSMMUG00000040348	HIST1H2BJ	1.02	3.87E−02	histone cluster 1, H2bj
ENSMMUG00000016585	DLG1	−0.51	3.87E−02	discs large MAGUK scaffold protein 1
ENSMMUG00000023676	ESPN	−1.20	3.96E−02	espin
ENSMMUG00000022161	CA11	−0.45	3.96E−02	carbonic anhydrase 11
ENSMMUG00000021448		−0.55	3.96E−02	
ENSMMUG00000016698	PNPLA3	−0.55	4.04E−02	patatin like phospholipase domain containing 3
ENSMMUG00000001814	LMCD1	−1.15	4.04E−02	LIM and cysteine rich domains 1
ENSMMUG00000020027	PGAM1	−0.35	4.04E−02	phosphoglycerate mutase 1
ENSMMUG00000043141		−2.24	4.06E−02	#N/A
ENSMMUG00000006475	CHST12	0.68	4.09E−02	carbohydrate sulfotransferase 12
ENSMMUG00000016715	PIDD1	−0.83	4.09E−02	p53−induced death domain protein 1
ENSMMUG00000004843	SCN1B	0.51	4.09E−02	sodium voltage−gated channel beta subunit 1
ENSMMUG00000041930	TMEM38A	0.60	4.09E−02	#N/A
ENSMMUG00000038997	ZAR1L	−13.30	4.09E−02	zygote arrest 1 like
ENSMMUG00000040135	PDCL3	−0.43	4.10E−02	phosducin like 3
ENSMMUG00000005263	CXorf38	0.29	4.12E−02	chromosome X CXorf38 homolog
ENSMMUG00000015892	CSNK2A2	0.56	4.15E−02	casein kinase 2 alpha 2
ENSMMUG00000040897		13.25	4.16E−02	#N/A
ENSMMUG00000000706	FLNB	−0.48	4.16E−02	filamin B
ENSMMUG00000014932	MEGF8	0.48	4.17E−02	multiple EGF like domains 8
ENSMMUG00000021165	RHOBTB3	−0.42	4.17E−02	Rho related BTB domain containing 3
ENSMMUG00000003909	FTL	0.57	4.17E−02	ferritin light chain
ENSMMUG00000017423	RBM10	0.33	4.20E−02	RNA binding motif protein 10
ENSMMUG00000004963	LXN	0.85	4.21E−02	latexin
ENSMMUG00000044603	ZFP36L1	−0.36	4.22E−02	ZFP36 ring finger protein like 1
ENSMMUG00000010446	ZNF330	−0.39	4.22E−02	#N/A
ENSMMUG00000018119	SNX19	0.24	4.25E−02	sorting nexin 19
ENSMMUG00000001250	PTPN3	0.36	4.29E−02	protein tyrosine phosphatase non−receptor type 3
ENSMMUG00000004087	BRAT1	0.32	4.30E−02	BRCA1 associated ATM activator 1
ENSMMUG00000014163	TICAM1	0.71	4.31E−02	toll like receptor adaptor molecule 1
ENSMMUG00000016921	CYTL1	−0.71	4.34E−02	cytokine like 1
ENSMMUG00000000208	NRG1	−1.13	4.34E−02	neuregulin 1

ENSMMUG00000016522	NLN	−0.41	4.35E−02	neurolysin
ENSMMUG00000016167	PTGER4	−0.94	4.35E−02	prostaglandin E receptor 4
ENSMMUG00000005856	GATM	−0.56	4.35E−02	glycine amidinotransferase
ENSMMUG00000000296	STAC2	−13.19	4.35E−02	SH3 and cysteine rich domain 2
ENSMMUG00000010032	EDEM2	0.46	4.36E−02	ER degradation enhancing alpha−mannosidase like protein 2
ENSMMUG00000000996	OXSR1	−0.33	4.36E−02	oxidative stress responsive kinase 1
ENSMMUG00000021429	PTGDS	−0.43	4.36E−02	#N/A
ENSMMUG00000045584	LUZP1	0.42	4.40E−02	leucine zipper protein 1
ENSMMUG00000040973	LRRC24	0.60	4.45E−02	leucine rich repeat containing 24
ENSMMUG00000021162	CDS1	1.26	4.46E−02	CDP−diacylglycerol synthase 1
ENSMMUG00000044290		−0.42	4.47E−02	#N/A
ENSMMUG00000017164	FRMD4A	−0.40	4.48E−02	FERM domain containing 4A
ENSMMUG00000012432	LRRC17	−0.78	4.48E−02	leucine rich repeat containing 17
ENSMMUG00000022222	IL17RA	0.32	4.49E−02	interleukin 17 receptor A
ENSMMUG00000040761		13.11	4.49E−02	#N/A
ENSMMUG00000023765	DNMBP	0.42	4.49E−02	dynamin binding protein
ENSMMUG00000011512	ANO2	−0.74	4.50E−02	#N/A
ENSMMUG00000044472	DDAH2	−0.49	4.50E−02	dimethylarginine dimethylaminohydrolase 2
ENSMMUG00000003669	MSRB3	−0.32	4.50E−02	methionine sulfoxide reductase B3
ENSMMUG00000022467	MBTPS2	0.59	4.53E−02	membrane bound transcription factor peptidase, site 2
ENSMMUG00000019423	ZNF2	0.58	4.54E−02	zinc finger protein 2
ENSMMUG00000000453	SPPL2B	0.41	4.59E−02	signal peptide peptidase−like 2B
ENSMMUG00000009383	CC2D2A	−0.53	4.61E−02	coiled−coil and C2 domain containing 2A
ENSMMUG00000022677	GJB3	−2.05	4.61E−02	gap junction protein beta 3
ENSMMUG00000042349	IFI27L2	−0.36	4.61E−02	#N/A
ENSMMUG00000044304	AASS	−0.56	4.62E−02	aminoadipate−semialdehyde synthase
ENSMMUG00000006527	CALD1	−0.65	4.62E−02	caldesmon 1
ENSMMUG00000045509	NAALADL2	0.79	4.62E−02	N−acetylated alpha−linked acidic dipeptidase like 2
ENSMMUG00000041160	ZDHHC18	0.57	4.64E−02	#N/A
ENSMMUG00000016096	LMNA	−0.36	4.65E−02	lamin A/C
ENSMMUG00000008487	NQO2	0.39	4.65E−02	N−ribosyldihydronicotinamide:quinone reductase 2
ENSMMUG00000008934	MARCKS	−0.23	4.68E−02	myristoylated alanine rich protein kinase C substrate
ENSMMUG00000048072	FKBP2	−0.28	4.69E−02	FKBP prolyl isomerase 2
ENSMMUG00000009617	PTPN18	0.45	4.69E−02	protein tyrosine phosphatase non−receptor type 18
ENSMMUG00000020861	EVC	0.30	4.72E−02	EvC ciliary complex subunit 1
ENSMMUG00000016508	C9orf72	−0.58	4.73E−02	chromosome 15 C9orf72 homolog
ENSMMUG00000012100	CDH15	−1.22	4.74E−02	cadherin 15
ENSMMUG00000008790	ZMIZ1	0.60	4.75E−02	zinc finger MIZ−type containing 1

ENSMMUG00000015708	ANXA7	−0.19	4.79E−02	annexin A7
ENSMMUG00000016074	LFNG	1.06	4.80E−02	LFNG O−fucosylpeptide 3−beta−N−acetylglucosaminyltransferase
ENSMMUG00000011245	CRISPLD2	−0.76	4.82E−02	cysteine rich secretory protein LCCL domain containing 2
ENSMMUG00000001641	NAPRT	−0.44	4.82E−02	nicotinate phosphoribosyltransferase
ENSMMUG00000005547	SF3A1	0.34	4.88E−02	splicing factor 3a subunit 1
ENSMMUG00000001473	CNN1	−2.28	4.90E−02	calponin 1
ENSMMUG00000014465	H2AFY	−0.23	4.91E−02	H2A histone family, member Y
ENSMMUG00000005199	LPCAT1	0.60	4.94E−02	lysophosphatidylcholine acyltransferase 1
ENSMMUG00000000556	HAPLN3	−0.47	4.95E−02	hyaluronan and proteoglycan link protein 3
ENSMMUG00000043967		−1.55	4.96E−02	#N/A
ENSMMUG00000013290	LURAP1L	−0.93	4.96E−02	leucine rich adaptor protein 1 like
ENSMMUG00000006770	C7orf55−LUC7L2	−0.26	5.00E−02	putative RNA−binding protein Luc7−like 2

Table S2. GO categories of DEGs in mRPE cells after SA treatment.

GO Id	Description	q−value
GO:0030198	extracellular matrix organization	3.90E−07
GO:0031012	extracellular matrix	3.90E−07
GO:0030048	actin filament−based movement	3.90E−07
GO:0043062	extracellular structure organization	3.90E−07
GO:0022617	extracellular matrix disassembly	8.23E−07
GO:0005578	proteinaceous extracellular matrix	4.51E−06
GO:0070252	actin−mediated cell contraction	6.52E−06
GO:0003012	muscle system process	1.12E−05
GO:0006936	muscle contraction	1.33E−05
GO:0015629	actin cytoskeleton	2.84E−05
GO:0006695	cholesterol biosynthetic process	4.60E−05
GO:0044420	extracellular matrix part	5.40E−05
GO:0016126	sterol biosynthetic process	5.56E−05
GO:0050673	epithelial cell proliferation	6.68E−04
GO:0006066	alcohol metabolic process	7.43E−04
GO:0005581	collagen	8.58E−04
GO:0030574	collagen catabolic process	1.51E−03
GO:1901617	organic hydroxy compound biosynthetic process	1.84E−03
GO:0016125	sterol metabolic process	2.24E−03
GO:0008203	cholesterol metabolic process	2.24E−03

GO:0044243	multicellular organismal catabolic process	3.41E−03
GO:0050678	regulation of epithelial cell proliferation	3.66E−03
GO:0032963	collagen metabolic process	5.43E−03
GO:0034329	cell junction assembly	5.43E−03
GO:0001935	endothelial cell proliferation	5.43E−03
GO:1901681	sulfur compound binding	5.52E−03
GO:0001501	skeletal system development	6.03E−03
GO:0046165	alcohol biosynthetic process	6.03E−03
GO:0061061	muscle structure development	6.90E−03
GO:0044449	contractile fiber part	6.90E−03
GO:0044259	multicellular organismal macromolecule metabolic process	6.90E−03
GO:0050680	negative regulation of epithelial cell proliferation	7.65E−03
GO:0007517	muscle organ development	7.69E−03
GO:0030049	muscle filament sliding	8.19E−03
GO:0033275	actin−myosin filament sliding	8.19E−03
GO:0007519	skeletal muscle tissue development	9.54E−03
GO:0036379	myofilament	1.04E−02
GO:0044236	multicellular organismal metabolic process	1.19E−02
GO:0018108	peptidyl−tyrosine phosphorylation	1.22E−02
GO:0018212	peptidyl−tyrosine modification	1.22E−02
GO:0006631	fatty acid metabolic process	1.22E−02
GO:0050730	regulation of peptidyl−tyrosine phosphorylation	1.26E−02
GO:0008201	heparin binding	1.29E−02
GO:0034330	cell junction organization	1.32E−02
GO:0030017	sarcomere	1.34E−02
GO:0005583	fibrillar collagen	1.36E−02
GO:0016053	organic acid biosynthetic process	1.36E−02
GO:0046394	carboxylic acid biosynthetic process	1.36E−02
GO:0043292	contractile fiber	1.36E−02
GO:0048741	skeletal muscle fiber development	1.36E−02
GO:0003013	circulatory system process	1.37E−02
GO:0014706	striated muscle tissue development	1.47E−02
GO:0016323	basolateral plasma membrane	1.49E−02
GO:0030279	negative regulation of ossification	1.55E−02
GO:0001936	regulation of endothelial cell proliferation	1.58E−02
GO:0007178	transmembrane receptor protein serine/threonine kinase signaling pathway	1.59E−02
GO:0019838	growth factor binding	1.80E−02

GO:0001525	angiogenesis	1.92E−02
GO:0014904	myotube cell development	1.98E−02
GO:0050795	regulation of behavior	2.06E−02
GO:0006694	steroid biosynthetic process	2.48E−02
GO:0060537	muscle tissue development	2.48E−02
GO:0005865	striated muscle thin filament	2.67E−02
GO:0070168	negative regulation of biomineral tissue development	2.67E−02
GO:0060538	skeletal muscle organ development	2.67E−02
GO:0040017	positive regulation of locomotion	2.76E−02
GO:0008015	blood circulation	2.76E−02
GO:0071559	response to transforming growth factor beta	3.12E−02
GO:0071560	cellular response to transforming growth factor beta stimulus	3.12E−02
GO:0016616	oxidoreductase activity, acting on the CH−OH group of donors, NAD or NADP as acceptor	3.22E−02
GO:0006023	aminoglycan biosynthetic process	3.35E−02
GO:0050731	positive regulation of peptidyl−tyrosine phosphorylation	3.63E−02
GO:1901605	alpha−amino acid metabolic process	3.63E−02
GO:0051146	striated muscle cell differentiation	3.91E−02
GO:0001937	negative regulation of endothelial cell proliferation	3.91E−02
GO:0005539	glycosaminoglycan binding	3.93E−02
GO:0060627	regulation of vesicle−mediated transport	4.10E−02
GO:0050920	regulation of chemotaxis	4.27E−02
GO:0022803	passive transmembrane transporter activity	4.28E−02
GO:0015267	channel activity	4.28E−02
GO:0030016	myofibril	4.35E−02
GO:0090257	regulation of muscle system process	4.35E−02
GO:0006790	sulfur compound metabolic process	4.53E−02
GO:0051272	positive regulation of cellular component movement	4.66E−02
GO:0001568	blood vessel development	4.66E−02
GO:0007160	cell−matrix adhesion	4.66E−02

Table S3. DEGs in mRPE cells after CAY10556 treatment.

Ensembl ID	GeneID	log2FoldChange	Padj	Gene Description
ENSMMUG00000002038	CEMIP	0.45	1.57E−06	cell migration inducing hyaluronidase 1
ENSMMUG00000003589	ANK3	−0.76	8.88E−06	ankyrin 3
ENSMMUG00000000706	FLNB	0.38	4.45E−05	filamin B

ENSMMUG00000058462	SCD	0.48	4.54E−05	stearoyl−CoA desaturase
ENSMMUG00000008247	MXRA5	0.59	7.21E−05	matrix remodeling associated 5
ENSMMUG00000013446	ANXA8	−0.44	3.15E−04	annexin A8
ENSMMUG00000019082	SFRP1	0.37	3.15E−04	secreted frizzled related protein 1
ENSMMUG00000001328	ALDH3A1	−0.42	6.82E−04	aldehyde dehydrogenase 3 family member A1
ENSMMUG00000046124	SLC2A3	−0.54	6.94E−04	solute carrier family 2 (facilitated glucose transporter), member 3
ENSMMUG00000010200	NDRG1	−0.58	7.81E−04	N−myc downstream regulated 1
ENSMMUG00000012054	ACTG1	−0.27	1.14E−03	actin gamma 1
ENSMMUG00000008702	SYNPO2	0.34	1.14E−03	synaptopodin 2
ENSMMUG00000056699	BHLHE40	0.41	1.34E−03	basic helix−loop−helix family member e40
ENSMMUG00000000327	FADS2	0.49	1.37E−03	fatty acid desaturase 2
ENSMMUG00000013057	ALDH1B1	0.45	2.11E−03	aldehyde dehydrogenase 1 family member B1
ENSMMUG00000000777	DST	−0.29	4.24E−03	dystonin
ENSMMUG00000014454	SOX4	0.45	4.29E−03	SRY−box transcription factor 4
ENSMMUG00000015851	SFRP2	0.40	6.89E−03	secreted frizzled related protein 2
ENSMMUG00000013945	CYP1B1	−0.95	7.64E−03	cytochrome P450, family 1, subfamily B, polypeptide 1
ENSMMUG00000041480	IGFBP5	0.24	1.11E−02	insulin like growth factor binding protein 5
ENSMMUG00000023248	STK38L	0.39	1.16E−02	serine/threonine kinase 38 like
ENSMMUG00000005136	TCAF1	−0.39	1.16E−02	TRPM8 channel associated factor 1
ENSMMUG00000016872	EFEMP1	0.24	1.17E−02	EGF containing fibulin extracellular matrix protein 1
ENSMMUG00000005220	SYNM	0.30	1.29E−02	synemin
ENSMMUG00000006340	SLC6A6	0.48	1.30E−02	solute carrier family 6 member 6
ENSMMUG00000002368	CCND1	0.26	1.60E−02	cyclin D1
ENSMMUG00000020953	MICAL2	0.27	1.91E−02	F−actin−monooxygenase MICAL2
ENSMMUG00000003755	ITGA5	0.29	1.96E−02	integrin subunit alpha 5
ENSMMUG00000003638	LOXL1	0.33	2.01E−02	lysyl oxidase like 1
ENSMMUG00000015001	LIMK2	0.33	2.34E−02	LIM domain kinase 2
ENSMMUG00000009906	PLAU	0.34	2.34E−02	plasminogen activator, urokinase
ENSMMUG00000031024	LDHA	−0.29	2.79E−02	lactate dehydrogenase A
ENSMMUG00000011274	P4HA1	−0.41	3.21E−02	prolyl 4−hydroxylase subunit alpha 1
ENSMMUG00000007805	LIMCH1	−0.38	3.26E−02	LIM and calponin homology domains 1
ENSMMUG00000064405	MYH2	0.44	3.28E−02	myosin−8
ENSMMUG00000011245	CRISPLD2	0.58	3.38E−02	cysteine rich secretory protein LCCL domain containing 2
ENSMMUG00000015877	GBE1	−0.36	3.46E−02	1,4−alpha−glucan branching enzyme 1
ENSMMUG00000016803	SCRN1	−0.38	3.53E−02	secernin 1
ENSMMUG00000011952	PPM1H	0.32	3.54E−02	protein phosphatase, Mg2+/Mn2+ dependent 1H
ENSMMUG00000016939	OSBPL3	0.31	3.81E−02	oxysterol binding protein like 3
ENSMMUG00000009425	LOC705671	−0.23	3.94E−02	actin, cytoplasmic 2−like

ENSMMUG00000015314	TSPAN15	0.26	3.94E−02	tetraspanin 15
ENSMMUG00000052424	NA	−0.27	4.08E−02	
ENSMMUG00000010615	GALNT10	0.24	4.25E−02	polypeptide N−acetylgalactosaminyltransferase 10
ENSMMUG00000057432	STK17B	−0.43	4.35E−02	serine/threonine kinase 17b
ENSMMUG00000010767	ACTN1	0.21	4.99E−02	actinin alpha 1
ENSMMUG00000020772	CSRP1	0.21	4.99E−02	cysteine and glycine rich protein 1
ENSMMUG00000013546	GOLGB1	−0.30	4.99E−02	golgin B1
ENSMMUG00000003565	LGALS3	−0.36	4.99E−02	galectin 3
ENSMMUG00000003572	NAMPT	0.42	4.99E−02	nicotinamide phosphoribosyltransferase
ENSMMUG00000001587	TPM1	0.20	4.99E−02	tropomyosin 1
